# Lentzeacins A-E, New Bacterial-Derived 2,5- and 2,6-Disubstituted Pyrazines from a BGC-Rich Soil Bacterium *Lentzea* sp. GA3-008

**DOI:** 10.3390/molecules26237197

**Published:** 2021-11-27

**Authors:** Hong-Bing Liu, Jack R. Davison, Rahim Rajwani, Gengxiang Zhao, Shannon I. Ohlemacher, Robert D. O’Connor, Carole A. Bewley

**Affiliations:** Laboratory of Bioorganic Chemistry, National Institute of Diabetes and Digestive and Kidney Diseases, National Institutes of Health, Bethesda, MD 20892-0820, USA; lhb200858@icloud.com (H.-B.L.); jdavison@lifeminetx.com (J.R.D.); rahim.rajwani@nih.gov (R.R.); gengxiang.zhao@nih.gov (G.Z.); shannon.ohlemacher@nih.gov (S.I.O.); robert.oconnor@nih.gov (R.D.O.)

**Keywords:** natural products, genomics, structure elucidation, cyclic dipeptides, NRPS, heterocycles, actinomycetes, biosynthesis

## Abstract

Pyrazines (1,4-diazirines) are an important group of natural products that have tremendous monetary value in the food and fragrance industries and can exhibit a wide range of biological effects including antineoplastic, antidiabetic and antibiotic activities. As part of a project investigating the secondary metabolites present in understudied and chemically rich Actinomycetes, we isolated a series of six pyrazines from a soil-derived *Lentzea* sp. GA3-008, four of which are new. Here we describe the structures of lentzeacins A-E (**1**, **3**, **5** and **6**) along with two known analogues (**2** and **4**) and the porphyrin zincphyrin. The structures were determined by NMR spectroscopy and HR-ESI-MS. The suite of compounds present in *Lentzea* sp. includes 2,5-disubstituted pyrazines (compounds **2**, **4**, and **6**) together with the new 2,6-disubstituted isomers (compounds **1**, **3** and **5**), a chemical class that is uncommon. We used long-read Nanopore sequencing to assemble a draft genome sequence of *Lentzea* sp. which revealed the presence of 40 biosynthetic gene clusters. Analysis of classical di-modular and single module non-ribosomal peptide synthase genes, and cyclic dipeptide synthases narrows down the possibilities for the biosynthesis of the pyrazines present in this strain.

## 1. Introduction

Pyrazines (1,4-diazines) are ubiquitous aromatic nitrogen-containing heterocycles distributed widely in nature, and are found in plants, insects, and diverse microorganisms. Alkyl-substituted pyrazines are often volatile compounds and are responsible for some of the flavors and aromas of plants, essential oils, coffee and wine, to name only a few. Recent surveys have estimated a multibillion-dollar global market for pyrazines in the food and fragrance industries [1]. Pyrazines play important ecological roles by affecting the behavior of numerous species of ants and comprise an important class of antibiotics produced by fungi such as *Aspergillus* spp. [2]. Interestingly, bacteria from many genera, including *Pseudomonas, Mycobacterium*, and *Rhodococcus*, can use pyrazine derivatives (such as 2-hydroxy pyrazine, 2,3-diethyl-5-methyl pyrazine, 2,5-dimethyl pyrazine, and tetramethyl pyrazine) as carbon and nitrogen sources [3,4]. Importantly, natural and synthetic pyrazines have been associated with a wide range of biological effects, including antitubercular, anticancer, diuretic, antidiabetic, insecticidal and nematicidal activities [2,5,6]. Two of the best known examples of pyrazine-containing medicines include the chemotherapeutic bortezomib (Velcade^TM^) [5] and the antitubercular drug pyrazinamide.

**Figure 1:**
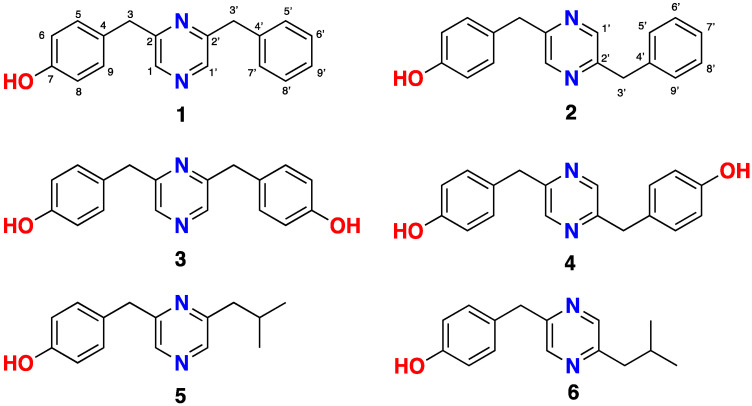
Lentzeacins A-E (**1**–**3**,**5**,**6**) and actinopolymorphol C (**4**) from *Lentzea* sp. GA3-008.

Due to their importance in agriculture and medicine and the prospect of engineering product diversity, independent groups are investigating the biosynthetic pathways that produce pyrazines and their amino acid-derived precursors including diketopiperazines and piperazines [7,8,9]. Recent studies have revealed several biosynthetic routes for the synthesis of 2,5-diketopiperazines (cyclic dipeptides). These include synthesis by cyclodipeptide synthetases (CDPS) that use aminoacyl-tRNAs (aa-tRNAs) for amino acid incorporation [7], and classical di-modular non-ribosomal peptide synthetases (NRPS) that produce compounds such as thaxtomin A [10] and gliotoxin in bacteria [11], and actinopolymorphol C [12], brasiliamide B [13] and hancockiamides in fungi [13,14]. Most recently single module NRPS-like genes containing a reductase domain have been shown to produce pyrazinones [15,16,17]. Among natural products, 2,5-disubstituted pyrazines are relatively common and their origins from cyclic dipeptides is obvious. In contrast, the occurrence of 2,6-disubstituted pyrazines is less common, thus far isolated from *Penicillium* spp. [18], myxobacteria [19] and a 2-keto pyrazine from enterohemorrhagic *Escherichia coli* [16]. Here we describe the isolation and structure determination of lentzeacins A-E (**1**–**3** and **5**,**6**) (Figure 1) from an understudied actinomycete strain *Lentzea* sp. GA3-008 where compounds **1**, **3**, **5** and **6** are new. Together with known compounds **2** and **4**, the porphyrin zincphyrin [20] was also isolated from strain GA3-008. Whole genome sequencing and analysis of the biosynthetic gene clusters (BGCs) in GA3-008 revealed the presence of a CDPS and a single module NRPS. The CDPS predicted product does not correspond to the lentzeacin structures, and the broad specificity of the NRPS adenylation domain precludes structure assignments leaving open the possibility that the lentzeacins are biosynthesized by a non-canonical NRPS or from non-clustered enzymes [6].

## 2. Results

### 2.1. Isolation and Structure Determination of Lentzeacins and Zincphyrin

*Lentzea* sp. GA3-008 was isolated from a desert soil sample on 10% actinomycete isolation agar and the axenic strain grown in liquid media for 10 days. The EtOAc extract of the culture broth inhibited the growth of *Staphylococcus aureus* ATCC 25913. Using antimicrobial assay-guided fractionation of the EtOAc extract and RP-HPLC, the active compound was identified as zincphyrin by high-resolution electrospray ionization mass spectrometry (HR-ESI-MS) and comparison of the ^13^C NMR data with published values [21]. Compounds **1**–**4** were readily detected by UV and MS in HPLC chromatograms and could be purified from the organic extract. Similar product ions were observed for compounds **1**–**4** in HR-MS/MS spectra (Figures S1–S7). Targeted analysis for these product ions in the total MS/MS data of the organic extract led to the identification of two additional pyrazines **5** and **6** that were purified by HPLC.

**Figure 2:**
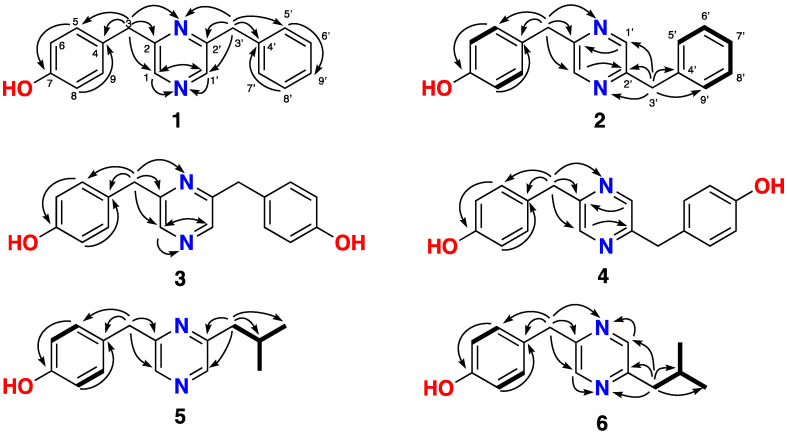
Key ^1^H-^1^H COSY (bold lines) and HMBC (^1^H-^13^C and ^1^H-^15^N) NMR (nuclear magnetic resonance) correlations (arrows) observed for compounds **1**–**6**.

Compound **1** was isolated as a light-yellow oil. The molecular formula, C_18_H_16_N_2_O with 12 degrees of unsaturation, was determined by HR-ESI-MS(+), which showed a peak at *m*/*z* 277.1345 ([M + H]^+^ calculated 277.1341). (See Figures S8–S13 for spectral data for compound **1**.) Only 14 resonances were observed in the ^13^C NMR spectra (Table 1) of **1**, indicating the presence of symmetry. The presence of a *para*-substituted phenyl ring (accounting for the element of symmetry) was indicated by two coupled aromatic doublets at δ_H_ 7.08 (d, *J* = 8.1 Hz, H-5/9) and 6.71 (d, *J* = 8.1 Hz, H-6/8) and two aromatic methine carbon signals at δ_C_ 157.3 (C-7) and δ_C_ 130.5 (C-4). HMBC correlations (Figure 2) from H-5/9 to C-7 and H-6/8 to C-4 indicated that this benzene ring was a 4-hydroxyphenyl group. A monosubstituted benzene ring was supported by the coupling pattern observed for three aromatic methine protons at δ_H_ 7.21 (t, *J* = 6.9 Hz, H-7′), 7.26 (d, *J* = 6.9 Hz, H-5′/9′) and 7.28 (d, *J* = 6.9 Hz, H-6′/8′) in **1**. The HMBC correlations from H_2_-3 to C-1, C-2, C-4, and C-5/9 and from H_2_-3′ to C-1′, C-2′, C-4′, and C-5′/9′ and the molecular formula indicated the 4-hydroxyphenyl group and monosubstituted benzene ring were linked to the pyrazine group via CH_2_ groups in **1**, respectively. The ^15^N-HMBC correlations from H_2_-3 and H_2_-3′ to 2-N (δ_N_ 327.7) and from H_2_-1 and H_2_-1′ to 1-N (δ_N_ 325.4) confirmed compound **1** was a rare 2,6-disubstituted pyrazine that was named lentzeacin A.

Compound **2** was isolated as a light-yellow oil. HR-ESI-MS(+) showed a pseudomolecular ion peak at *m*/*z* 277.1343, indicating it shared the same molecular formula of C_18_H_16_N_2_O ([M + H]^+^, calculated 277.1341) with compound **1** (Spectral data for compound **2** appear in Figures S14–S20.) The 1D NMR data of compounds **1** and **2** (Table 1) were almost superimposable. The HMBC correlations (Figure 2) from H_2_-3 to C-1 C-2, C-4, and C-5/9, from H_2_-3′ to C-1′, C-2′, C-4′, and C-5′/9′, from H-1 to C-2′, and from H-1′ to C-2 indicated compound **2** contains a 2,5-substituted pyrazine core structure that was supported by the ^15^N-HMBC correlations from H_2_-3 to 2-N (δ_N_ 334.0) and from H_2_-3′ to 1-N (δ_N_ 333.6). Compound **2** was previously detected by LC-MS from in vitro non-ribosomal peptide synthetase reactions [17] and in vivo heterologous expression of a non-ribosomal peptide synthetase in *Escherichia coli* [22]. Here, compound **2** was named lentzeacin B.

Compounds **3** and **4** were isolated as light-yellow oils with the same molecular formula of C_18_H_16_N_2_O_2_, as established from the HR-ESI-MS(+) ions at *m*/*z* 293.1290 ([M + H]^+^ calculated 293.1290) and 293.1295 ([M + H]^+^ calculated 293.1290), respectively (see Figures S21–S26 and S27–S31 for spectral data for compounds **3** and **4**, respectively). The ^13^C NMR spectra of **3** and **4** revealed seven unique carbon resonances and the ^1^H NMR spectra of **3** and **4** only showed five signals, indicating they are symmetrical. Analysis of the 2D NMR spectra of **3** and **4** (Figure 2) suggested the presence of a 4-hydroxyphenyl group, which indicated two 4-hydroxyphenyl groups were connected to the pyrazine group via a CH_2_. The ^15^N-HMBC correlations from H_2_-3/3′ to 2-N (δ_N_ 329.3) and from H_2_-1/1′ to 1-N (δ_N_ 336.3) confirmed compound **3** was a 2,6-disubstituted pyrazine and was named lentzeacin C. Similarly, the ^15^N-HMBC correlations from H_2_-3/3′ to 1-N/2-N (δ_N_ 333.0) implied compound **4** was a 2,5-disubstituted pyrazine. Compound **4** was previously isolated from *Actinopolymorpha rutilus* by Shen and co-workers in 2010 and named actinopolymorphol C [12].

Compounds **5** and **6** also had the same molecular formula, C_15_H_18_N_2_O, as determined by HR-ESI-MS(+) of the protonated molecules at *m*/*z* 243.1497 and 243.1496 ([M + H]^+^ calculated 243.1497), respectively. The 1D NMR spectra of **5** and **6** were very similar to those of **1** and **2** (see Figures S32–S44 for spectral data for compounds **5** and **6**). The benzene group in **1** and **2** is replaced by an isopropyl group, which was confirmed by the ^1^H-^1^H COSY correlations from H-3′ to H_3_-5′/6′ and the HMBC correlations from H-3′ to C-1′, C-2′, C-4′ and C-5′/6′ (Figure 2). The ^15^N-HMBC correlations between H_2_-3 and H-1′ to 2-N (δ_N_ 324.5) and H_2_-3′ and H-1 to 1-N (δ_N_ 326.3) implied compound **6** was a 2,5-disubstituted pyrazine compound and was named lentzeacin D. Compound **5** was a 2,6-disubstituted pyrazine compound and named lentzeacin E.

### 2.2. Analysis of the GA3-008 Genome for Pyrazine Biosynthesis

**Figure 3:**
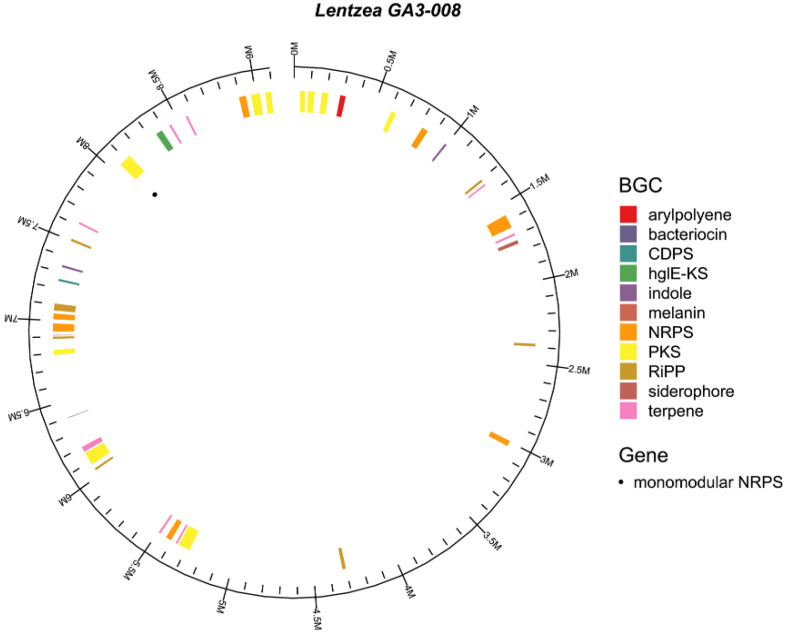
Genome map and biosynthetic gene clusters (BGCs) of *Lentzea* sp. GA3-008. The middle circle shows the presence of all BGCs in the *Lentzea* genome colored by compound type including a cyclodipeptide synthetase (CDPS, teal, 7–7.5 Mb) and a monomodular non-ribosomal peptide synthetase (NRPS, approximately 8 Mb). The outer most circle shows the approximate genomic position on a Mb scale.

Because the biosynthetic origins of 2,6-disubstituted pyrazines have never been determined for actinomycetes and this substitution pattern is rare, we carried out whole genome sequencing to look for potential biosynthetic gene clusters that could be connected to the lentzeacins. Biosynthetically, compounds **1**–**6** may be traced back to tyrosine, phenylalanine, and leucine. We used Nanopore Minion long-read sequencing to assemble a draft genome for *Lentzea* sp.GA3-008 and submitted the 9.19 Mb genome to the program antiSMASH to predict BGCs and potentially identify the biosynthetic pathway responsible for production of the 2,5- and 2,6-disubstituted pyrazines. Forty BGCs of varying types were predicted and included six terpenes, three NRPs and three NRPS-like proteins, seven ribosomally synthesized peptides (RiPPs), three polyketide synthase-like (PKS), two type-1 polyketide synthases (TIPKS), eight hybrid pathways, one T3PKS, two T2PKSs, and one of each of the CDPS, aminoglycoside, heterocyst glycolipid synthase-like PKS (hgIE-KS), and alkaloid BGCs (Figure 3 and Table S1) [23].

**Figure 4:**
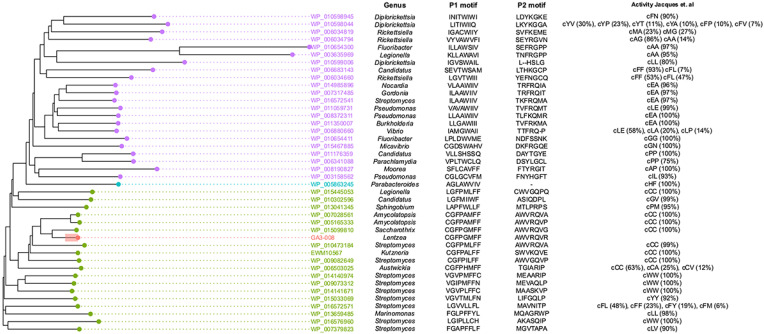
Specificity of CDPS (KOEHFPPE_07267) in *Lentzea* sp. GA3-008. Right panel shows phylogenetic tree of previously characterized CDPS with their accession numbers. The tree is colored according to the previously reported phylogenetic sub-families of CDPS (purple = XYP, green = NYH, blue = SYQ). KOEHFPPE_07267 from GA3-008 is highlighted in red. P1 and P2 motif corresponds to the amino acid residues in amino acyl t-RNA binding pocket one or two, respectively [9]. Activity data demonstrating amino acid specificity is derived from Jacques et al. [24].

From the antiSMASH output, there were two types of BGCs that could correspond to synthesis of the pyrazines, namely a CDPS and a monomodular NRPS. CDPSs recognize and reroute specific aa-tRNAs from ribosomal peptide synthesis to catalyze formation of cyclic dipeptides [24]. The specificity CDPSs from diverse genera for aa-tRNAs has been thoroughly characterized and reviewed by Jacques et al. [24]. Thus, specificity of a new CDPS can be predicted through comparison of residues in the aa-tRNA binding pockets, P1 and P2, and phylogenetic clustering [9]. The P1 and P2 binding pockets in the GA3-008 CDPS (gene KOEHFPPE_07267) have the respective amino acid sequences CGFPGMFF and AWVRQVR that are identical (for P1) or highly similar (for P2) to known CDPSs with specificity for Cys (Figure 4). If lentzeacins were to be synthesized by a CDPS, the binding pockets would be expected to recognize Phe, Tyr, or Ile. Therefore, KOEHFPPE_07267 is inconsistent with biosynthesis of lentzeacins.

Another route for pyrazine biosynthesis involves an uncommon monomodular NRPS with a terminal reductase domain (TD). Conversion of the amino acid to the reactive amino aldehyde allows for cyclization to form pyrazines [25,26,27,28]. The genome of *Lentzea* sp. GA3-008 harbors a single monomodular NRPS-like gene (KOEHFPPE_08075) possessing a terminal reductase domain (Figure S45). KOEHFPPE_08075 shares 50% amino acid identity to a previously characterized carboxylic acid reductase (CAR) in *Nocardia iowensis* (PDB ID: 5msc) [29]. CAR enzymes are NRPS-like and functionally related to monomodular NRPSs with TD domains. Both reduce carboxylic acids to corresponding aldehydes; however, the adenylation domains in CARs can have broad substrate selectivity [29,30]. AntiSMASH 5/NRPSPredictor2 could not predict specificity for the adenylation domain in KOEHFPPE_08075 (Table S2). KOEHFPPE_08076, a gene adjacent to the monomodular NRPS, is a homologue of Tyr-sensitive phospho-2-dehydro-3-deoxyheptonate aldolase (commonly annotated as *aroF*). *aroF* (EC:2.5.1.54) catalyzes the first step in the shikimate pathway for biosynthesis of Phe, Tyr and Trp. If KOEHFPPE_08075 is involved in lentzeacin biosynthesis, KOEHFPPE_08076 could provide control over supply of precursor aromatic amino acid substrates. KOEHFPPE_08076 is the second copy of *aroF* in the *Lentzea* sp. *GA3-008* genome. The other *aroF* copy is located the with enzymes of the shikimate pathway, as seen in other *Lentzea* sp. genomes (Figure S46). In BLAST searches against the NCBI non-redundant nucleotide sequence database, we did not find any other *Lentzea* sp. genome harboring a similar BGC. However, a taxonomically related strain *Allokutzneria albata* strain DSM 44149, also a member of the family Pseudonocardiaceae, encoded a near identical copy of this cluster (Figure S47).

## 3. Discussion

**Scheme 1:**
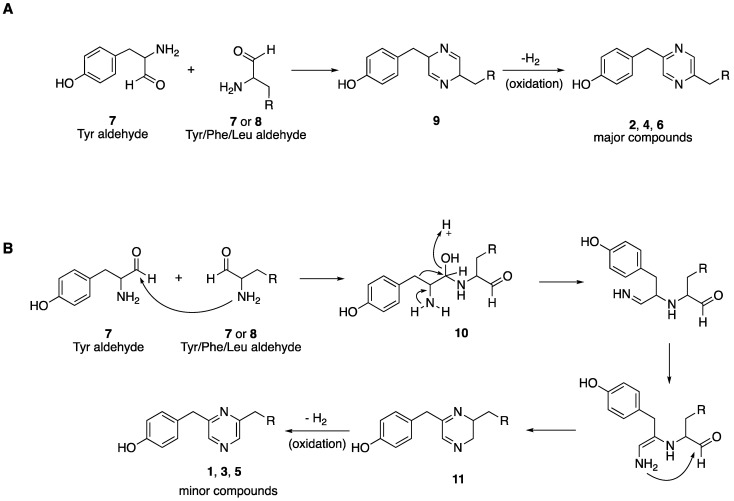
Biosynthetic proposal for (**A**) 2,5-disubstituted pyrazines **2**, **4**, and **6**, and (**B**) 2,6-disubstituted pyrazines **1**, **3**, and **5**.

Nanopore sequencing and analysis of a draft genome of GA3-008 facilitated a search for the biosynthetic pathway leading to the lentzeacins. While this analysis leaves open the possibility that the lentzeacins are synthesized via a monomodular NRPS-like enzyme, it does not explain the presence of the 2,6-disubstituted compounds that occur together with their 2,5-disubstituted congeners. To the best of our knowledge, the co-occurrence of such a pair of disubstituted piperazines has been observed in fungal-derived brasiliamides isolated from *Penicillium brasilianum* [18] and trace amounts in the myxobacterium *Chondromyces crocatus* [31]. Biosynthesis of the brasiliamides was originally proposed to occur via a phenylpropanoid pathway involving phenylalanine/tyrosine ammonia lyase (PAL) and *p*-coumaric acid because two units of [2-^13^C]-phenylalanine were incorporated in brasiliamide structures [18]. These enzymes were also detected in the *Lentzea* sp. GA3-008 genome (Figure 3). However, a recent study convincingly demonstrated that the biosynthesis of the diketopiperazine core of brasiliamides is catalyzed by a monomodular NRPS (*brs*) through stereoselective in vitro activity of a purified BrsA protein on its substrate L-phenylalanine [13]. Together, these analyses leave open the possibility that the 2,5-disubstituted pyrazines may be produced by the monomodular NRPS-like gene described above that would yield dihydropyrazine **9** followed by spontaneous oxidation to give the major compounds **2**, **4** and **6** (Scheme 1a). Alternatively, rearrangement of a reactive dipeptide aldehyde **10** (Scheme 1b) followed by cyclization would yield 2,6-disubstituted dihydropyrazine **11** followed by oxidation to give the minor metabolites **1**, **3** and **5**. Such a scheme has a precedent in the myxobacterium *C. crocatus* as demonstrated through incorporation of ^15^N_2_ Val in both isomers [19]. Further studies involving labeled amino acids or production and characterization of the product of the single module NRPS KOEHFPPE_08075 should prove useful in determining the biosynthesis of these unusual actinomycete-associated metabolites. Interestingly, of the 40 BGCs identified by antiSMASH 5.0, only two showed 100% similarity to BGCs for known compounds. These included a RiPP natural product citrulassin D and two copies of the terpene geosmin linked to BGCs 19 and 33 (Table S1). The low similarity of more than 30 additional clusters to known BGCs suggests GA3-008 is a promising source of novel secondary or specialized metabolites.

## 4. Materials and Methods

### 4.1. General Experimental Procedures

NMR spectra were measured on Bruker Avance 500 or 600 MHz spectrometers equipped with [^2^H, ^15^N, ^13^C, ^1^H], z-gradient cryoprobes. Chemical shifts (δ) were reported in parts per million (ppm) relative to the chemical shift of the solvent. LR-ESI-MS was carried out on an Agilent 1100 HPLC instrument and an Agilent 6310 Quadrupole mass spectrometer (Santa Clara, CA, USA). HR-ESI-MS was carried out on a Waters Time-of-Flight (TOF, Milford, MA, USA) mass spectrometer (Milford MA, USA) model LCT Premier or Agilent 6545 Q-TOF mass spectrometer. Semi-preparative HPLC was performed on an Agilent 1100 HPLC equipped with a SymmetryPrep C18 column (Waters, 7 μm, 7.8 × 300 mm) or Phenomenex kintex biphenyl column (5 uM, 100 Å, 10 × 250 mm). All solvents used for HPLC were of HPLC grade (Sigma Aldrich, St. Louis, MO, USA).

### 4.2. Isolation of Lentzea sp. GA3-008

A soil sample from Nevada (0.1 g) was heated in a 70 °C oven for 30 min and re-suspended in 0.5 mL of autoclaved water in a 15 mL polypropylene tube and vortexed for 1 min. The cells were allowed to settle at room temperature (rt) for 10 min. An aliquot of the suspension was diluted 10-fold with sterile water and 200 μL of the sample was plated on 10% actinomycete isolation agar (AIA with 1% agar) and stored for 1–2 weeks at rt. Actinomycete colonies appeared between one and two weeks and upon observation of branched hyphae or sporulation, 500 individual strains were re-streaked on 10% AIA plates to assess their purity. The amplified 16S rRNA gene fragment (1457 bp) displayed 99.44% identity to that of *Lentzea violacea* strain 173308 (GeneBank accession number EU570363.1) using a nucleotide BLASTsearch (nBLAST). The search also returned two strains of *Saccharothrix* spp., sj68 and LM062, as having 99.16% identity to the 16S gene (GeneBank accession numbers JX013968.1 and AF328674.1). To further probe the taxonomy, we computed an average nucleotide identity (ANI) for publicly available *Lentzea* and *Saccharothix* genomes. The mean ANI between GA3-008 and *Lentzea* genomes is 88.6% compared to 80.1% for *Saccharothrix* spp., confirming a closer taxonomic relationship to *Lentzea* (Figure S48). The 16S rRNA sequence is provided in the Supporting Information.

### 4.3. Solid Agar Screening for Antimicrobial Activity of Actinomycete Colonies

Actinomycete isolates growing on 10% AIA plates following re-streaking were screened for antimicrobial activity using an agar overlay assay (adapted from Maricic and Dawid [32]) where TSA agar (50 °C) inoculated with *Staphylococcus aureus* ATCC 25913 or *Escherichia coli* ATCC 25922 at a density of 10^4^ CFU/mL was slowly poured over the colonies. Plates were incubated at 37 °C overnight and zones of inhibition of growth of *E. coli* or *S. aureus* were visualized by eye. Colonies of strain GA3-008 inhibited the growth of *S. aureus*.

### 4.4. Genome Sequencing

High molecular weight genomic DNA was isolated from strain GA3-008 using a Wizard Genomic DNA Purification Kit (Promega, Madison, WI USA). Genomic DNA (1 µg) was sheared to a target MW of 20 kb using a gTube (Covaris, Woburn, MA USA), prepared for sequencing using the SQK-LSK109 genomic DNA by ligation kit (Oxford Nanopore, Oxford, UK), and sequenced on a FLO-MIN106D (r9.4.1) flow cell (Oxford Nanopore). Sequencing data were base-called using Guppy (v3.0.3, GPU-enabled high accuracy model, Oxford Nanopore), assembled using Canu [33] (v1.8), and polished using Medaka (v0.6.4, Oxford Nanopore) to create the final assembly. Gene models were predicted using Prokka [34] (v1.13) and BGCs were analyzed with antiSMASH (v5.0) [23]. The raw sequencing data (fastq files) for *Lentzea* sp. GA3-008 have been deposited to NCBI and are available under Project PRJNA769053, BioSample accession SAMN22082104. The assembled genome sequence is available at https://doi.org/10.6084/m9.figshare.16752457.v1 (accessed on 8 November 2021).

### 4.5. Fermentation, Extraction and Isolation

Strain GA3-008 was grown in 1 L actinomycetes broth vegitone (Sigma-Aldrich Cat. No. 40834-500G-F) for 10 days at 37 °C while shaking at 200 rpm. The culture broth was partitioned with the same volume of ethyl acetate followed by *n*-butanol. The organic layers were dried under reduced pressure to afford 0.8 g and 3.5 g of extract, respectively.

The EtOAc fraction was separated by semi-preparative HPLC (flowrate of 3 mL/min; 0–2 min, 5% B; 2–30 min gradient of 5–100% B; solvent A is 0.1% aqueous trifluoracetic acid and solvent B is MeCN) on a SymmetryPrep C18 column to yield 25 fractions (F1–F25). F20 was identified as zincphyrin by HR-ESI-MS and NMR. F15 was purified by semi-prep HPLC (flowrate of 3 mL/min, 0–20 min, 30–70% solvent B as above) on a Phenomenex Kintex biphenyl column to yield compounds **3** (0.3 mg, *t*_R_ 9.3 min) and **4** (0.8 mg, *t*_R_ 9.0 min). Using the same column, F19 was separated by semi-preparative HPLC (flowrate of 4 mL/min flow rate, 0–2 min, 35–35%; 2–30 min 35–50% MeCN/H_2_O gradient elution with 0.1% TFA), to yield compounds **1** (0.3 mg, *t*_R_ 16.7 min), **2** (1.9 mg, *t*_R_ 16.0 min), **5** (0.1 mg, *t*_R_ 14.6 min), and **6** (0.2 mg, *t*_R_ 14.0 min).

Lentzeacin A (**1**): Light-yellow oil; HR-ESIMS (positive ion) *m*/*z* 277.1345 (calculated for C_18_H_17_N_2_O [M + H]^+^, 277.1341); ^1^H NMR and ^13^C NMR, see Table 1.

Lentzeacin B (**2**): Light-yellow oil; HR-ESIMS (positive ion) *m*/*z* 277.1343 (calculated for C_18_H_17_N_2_O [M + H]^+^, 277.1341); ^1^H NMR and ^13^C NMR, see Table 1.

Lentzeacin C (**3**): Light-yellow oil; HR-ESIMS (positive ion) *m*/*z* 293.1290 (calculated for C_18_H_17_N_2_O_2_ [M + H]^+^, 293.1290); ^1^H NMR and ^13^C NMR, see Table 1.

Lentzeacin D (**5**): Light-yellow oil; HR-ESIMS (positive ion) *m*/*z* 243.1497 (calculated for C_15_H_19_N_2_O [M + H]^+^, 243.1497); ^1^H NMR and ^13^C NMR, see Table 1.

Lentzeacin E (**6**): Light-yellow oil; HR-ESIMS (positive ion) *m*/*z* 243.1496 (calculated for C_15_H_19_N_2_O [M + H]^+^, 243.1497); ^1^H NMR and ^13^C NMR, see Table 1.

### 4.6. Antimicrobial Assays

In the early stages of this work, we monitored the appearance of antimicrobial activity by culturing GA3-008 in 50 mL volumes of actinomycetes broth vegitone media for 10 days. Each day 500 μL aliquots were removed and partitioned with 500 μL EtOAc followed by 500 μL *n*-BuOH. We tested 20 μL aliquots of each organic extract in a solid agar 96-well pin assay where the agar was inoculated with the test strains. Test plates were incubated overnight at 37 °C and zones of inhibition were observed the next day. Antimicrobial activity as a function of time is shown in Figure S49. Antimicrobial screening of the mixtures in each of the Fractions 1–25 was performed using the same solid agar assay. Antimicrobial activity of compounds **1**–**6** and zincphyrin were tested in liquid broth in duplicate against *S. aureus* ATCC 29213 following the Clinical and Laboratory Standards Institute guidelines with the modification that the highest concentration tested was 200 μM [35]. Zincphyrin was the only compound that showed antimicrobial activity against *S. aureus* and it was not pursued further.

## Figures and Tables

**Table 1:** ^1^H and ^13^C NMR data of compounds **1**–**6**.

	1 ^A^		2 ^A^		3 ^B^		4 ^B^		5 ^C^		6 ^C^	
	δ_C_	δ_H_, multi. (J in Hz)	δ_C_	δ_H_, multi. (J in Hz)	δ_C_	δ_H_, multi. (J in Hz)	δ_C_	δ_H_, multi. (J in Hz)	δ_C_	δ_H_, multi. (J in Hz)	δ_C_	δ_H_, multi. (J in Hz)
1	142.7	8.27 (s)	144.7	8.38 (s)	141.6	8.33 (s)	143.3	8.43 (s)	142.5	8.26 (s)	144.6	8.37 (s)
2	158.1		156.1		155.7		154.0		157.8 ^D^		155.8	
3	41.6	4.06 (s, 2H)	41.1	4.04 (s, 2H)	40.7	3.96 (s, 2H)	39.6	3.94 (s, 2H)	41.6	4.05 (s, 2H)	41.1	4.05 (s, 2H)
4	130.5		130.5		129.0		129.1		130.6		130.6	
5	131.0	7.08 (d, 8.1)	131.0	7.07 (d, 8.4)	129.8	7.06 (d, 8.4)	129.8	7.04 (d, 8.5)	130.9	7.08 (d, 8.5)	131.0	7.08 (d, 8.5)
6	116.4	6.71 (d, 8.1)	116.5	6.71 (d, 8.4)	115.3	6.66 (d, 8.4)	115.3	6.66 (d, 8.5)	116.5	6.72 (d, 8.5)	116.5	6.72 (d, 8.5)
7	157.28		157.3		155.9		155.8		157.1 ^D^		157.3	
8	116.4	6.71 (d, 8.1)	116.5	6.71 (d, 8.4)	115.3	6.66 (d, 8.4)	115.3	6.66 (d, 8.5)	116.5	6.72 (d, 8.5)	116.5	6.72 (d, 8.5)
9	131.0	7.08 (d, 8.1)	131.0	7.07 (d, 8.4)	129.8	7.06 (d, 8.4)	129.8	7.04 (d, 8.5)	130.9	7.08 (d, 8.5)	131.0	7.08 (d, 8.5)
1′	142.6	8.29 (s)	144.6	8.40 (s)	141.6	8.33 (s)	143.3	8.43 (s)	143.0	δ 8.28 (s)	145.0	8.38 (s)
2′	157.34		155.3		155.7		154.0		157.5 ^D^		155.5	
3′	42.3	4.16 (s, 2H)	41.9	4.14 (s, 2H)	40.7	3.96 (s, 2H)	39.6	3.94 (s, 2H)	45.0	2.68 (d, 7.3, 2H)	44.6	2.66 (d, 7.2, 2H)
4′	139.8		139.9		129.0		129.1		30.3	2.10 (m)	30.3	2.05 (m)
5′	130.0	7.26 (d, 6.9)	130.0	7.25 (d, 7.0)	129.8	7.06 (d, 8.4)	129.8	7.04 (d, 8.5)	22.6	0.93 (d, 6.6, 3H)	22.6	0.93 (d, 6.6, 3H)
6′	129.7	7.28 (t, 6.9)	129.8	7.28 (t, 7.0)	115.3	6.66 (d, 8.4)	115.3	6.66 (d, 8.5)	22.6	0.93 (d, 6.6, 3H)	22.6	0.93 (d, 6.6, 3H)
7′	127.7	7.21 (t, 6.9)	127.7	7.20 (t, 7.0)	155.9		155.8					
8′	129.7	7.28 (t, 6.9)	129.8	7.28 (t, 7.0)	115.3	6.66 (d, 8.4)	115.3	6.66 (d, 8.5)				
9′	130.0	7.26 (d, 6.9)	130.0	7.25 (d, 7.0)	129.8	7.06 (d, 8.4)	129.8	7.04 (d, 8.5)				
N-1 ^E^	325.4		333.6		336.3		333.0				326.3	
N-2 ^E^	326.7		334.0		329.3		333.0				324.5	
-OH						9.25 (s)		9.23 (s)				

^A 1^H and ^13^C NMR were recorded in CD_3_OD at 500 and 125 MHz and referenced to residual solvent at δ_H_ 3.31 and δ_C_ 49.15 ppm, respectively; ^B 1^H and ^13^C NMR were recorded in DMSO-*d*_6_ at 500 and 125 MHz and referenced to residual solvent at δ_H_ 2.50 and δ_C_ 39.5 ppm, respectively; ^C 1^H and ^13^C NMR were recorded in CD_3_OD at 600 and 150 MHz and referenced to residual solvent at δ_H_ 3.31 and δ_C_ 49.15 ppm, respectively; ^D^ Chemical shifts are assigned from the HMBC spectra; ^E^ 600 MHz NMR: ^15^N, 0 ppm = 60.810645 MHz and ^1^H, 0 ppm = 600.13 MHz; 500 MHz NMR: ^15^N,0 ppm = 50.674693 MHz and ^1^H, 0 ppm = 500.1 MHz.

## Data Availability

The data presented in this study are available within the article, the Supplementary Materials, NCBI and FigShare.

## Supplementary Materials

The following are available online. 16S rRNA sequence, Figures S1–S49 showing 1D and 2D NMR, HR-MS and MS/MS spectra of compounds **1–6**, and BGCs and used in this study; Tables S1 and S2 of BGCs and adenylation domain specificity predictions for monomodular NRPS-like genes; Figures S4 and S45–S47 showing BGC comparisons and bioinformatics analyses described in this study.
