# Supplementary material for: Lentzeacins A-E, New Bacterial-Derived 2,5- and 2,6-Disubstituted Pyrazines from a BGC-Rich Soil Bacterium Lentzea sp. GA3-008
Source: Molecules. 2021 Nov 27;26(23):7197. doi: 10.3390/molecules26237197 (PMC8658869; doi:10.3390/molecules26237197)
Supplement: Supplementary file 1 [file molecules-26-07197-s001.zip › molecules-1477668 SI.pdf]

Supporting Information

Lentzeacins A-E, bacterial-derived 2,5- and 2,6-disubstituted pyrazines from a BGC-rich Soil Bacterium *Lentzea* sp. GA3-008

Hong-Bing Liu, Jack R. Davison, Rahim Rajwani, Gengxiang Zhao, Shannon I. Ohlemacher, Robert D. O'Connor, Carole A. Bewley*

Laboratory of Bioorganic Chemistry, National Institute of Diabetes and Digestive and Kidney Diseases, National Institutes of Health, Bethesda, Maryland 20892-0820, USA

Table of Contents

16S rRNA sequence
Figure S1. Structures and proposed fragmentation pathways of compounds **1-6**
Figure S2. HR-ESIMS/MS spectrum of **1** in positive mode
Figure S3. HR-ESIMS/MS spectrum of **2** in positive mode
Figure S4. HR-ESIMS/MS spectrum of **3** in positive mode
Figure S5. HR-ESIMS/MS spectrum of **4** in positive mode
Figure S6. HR-ESIMS/MS spectrum of **5** in positive mode
Figure S7. HR-ESIMS/MS spectrum of **6** in positive mode
Figure S8. ^{1}H NMR spectrum of **1** in methanol-d_4 at 298 K
Figure S9. ^{13}C NMR spectrum of **1** in methanol-d_4 at 298 K
Figure S10. HSQC spectrum of **1** in methanol-d_4 at 298 K
Figure S11. HMBC spectrum of **1** in methanol-d_4 at 298 K
Figure S12. ^{15}N-HMBC spectrum of **1** in methanol-d_4 at 298 K
Figure S13. HRESIMS spectrum of **1**
Figure S14. ^{1}H NMR spectrum of **2** in methanol-d_4 at 298 K
Figure S15. ^{13}C NMR spectrum of **2** in methanol-d_4 at 298 K
Figure S16. ^{1}H-^{1}H COSY spectrum of **2** in methanol-d_4 at 298 K
Figure S17. HSQC spectrum of **2** in methanol-d_4 at 298 K
Figure S18. HMBC spectrum of **2** in methanol-d_4 at 298 K
Figure S19. ^{15}N-HMBC spectrum of **2** in pyridine-d_4 at 298 K
Figure S20. HRESIMS spectrum of **2**
Figure S21. ^{1}H NMR spectrum of **3** in DMSO-d_6 at 298 K
Figure S22. ^{13}C NMR spectrum of **3** in DMSO-d_6 at 298 K
Figure S23. HSQC spectrum of **3** in DMSO-d_6 at 298 K
Figure S24. HMBC spectrum of **3** in DMSO-d_6 at 298 K
Figure S25. ^{15}N-HMBC spectrum of **3** in DMSO-d_6 at 298 K

* Corresponding author: Tel: +1-(301)-594-5187. E-mail: caroleb@mail.nih.gov

Figure S26. HRESIMS spectrum of **3**
Figure S27. ^{1}H NMR spectrum of **4** in DMSO-d_6 at 298 K
Figure S28. ^{13}C NMR spectrum of **4** in DMSO-d_6 at 298 K
Figure S29. HMBC spectrum of **4** in DMSO-d_6 at 298 K
Figure S30. ^{15}N-HMBC spectrum of **4** in DMSO-d_6 at 298 K
Figure S31. HRESIMS spectrum of **4**
Figure S32. ^{1}H NMR spectrum of **5** in methanol-d_4 at 298 K
Figure S33. ^{13}C NMR spectrum of **5** in methanol-d_4 at 298 K
Figure S34. ^{1}H-^{1}H COSY spectrum of **5** in methanol-d_4 at 298 K
Figure S35. HSQC spectrum of **5** in methanol-d_4 at 298 K
Figure S36. HMBC spectrum of **5** in methanol-d_4 at 298 K
Figure S37. HRESI-MS spectrum of **5**
Figure S38. ^{1}H NMR spectrum of **6** in methanol-d_4 at 298 K
Figure S39. ^{13}C NMR spectrum of **6** in methanol-d_4 at 298 K
Figure S40. ^{1}H-^{1}H COSY spectrum of **6** in methanol-d_4 at 298 K
Figure S41. HSQC spectrum of **6** in methanol-d_4 at 298 K
Figure S42. HMBC spectrum of **6** in methanol-d_4 at 298 K
Figure S43. ^{15}N-HMBC spectrum of **6** in methanol-d_4 at 298 K
Figure S44. HRESI-MS spectrum of **6**
Figure S45. BGC structure of monomodular NRPSs
Figure S46. AroF genes in *Lentzea* spp.
Figure S47. Monomodular NRPSs in *Lentzea* sp. and *Allokutzneria albata*
Figure S48. Plots of Average Nucleotide Identities for *Lentzea* spp. and *Saccharothrix* spp.
Figure S49. Time course of antimicrobial activity of cultures of GA3-008.

Table S1. Biosynthetic gene clusters predicted in *Lentzea sp*. identified by antiSMASH.
Table S2. Adenylation domain specificity predictions for monomodular NRPS-like genes (Excel file)

16S rRNA sequence

CCTTTGCCTTCGAGCTTACTATGCAAGTCGAGCGGTAAGGCCCTTCGGGGTACACGAG
CGGCGAACGGGTGAGTAACACGTGGGTAACCTGCCCTGTACTCTGGGATAAGCCTTGG
AAACGAGGTCTAATACCGGATACGACCTGGGATCGCATGATCTTGGGTGGAAAGTTCC
GGCGGTATGGGATGGACCCGCGGCCTATCAGCTTGTTGGTGGGGTAATGGCCTACCAA
GGCGACGACGGGTAGCCGGCCTGAGAGGGTGACCGGCCACACTGGGACTGAGACAC
GGCCCAGACTCCTACGGGAGGCAGCAGTGGGGAATATTGCACAATGGGCGAAAGCCT
GATGCAGCGACGCCGCGTGAGGGATGACGGCCTTCGGGTTGTAAACCTCTTTCAGCAG
GGACGAAGCGCAAGTGACGGTACCTGCAGAAGAAGCACCGGCTAACTACGTGCCAGCA
GCCGCGGTAATACGTAGGGTGCGAGCGTTGTCCGGATTTATTGGGCGTAAAGAGCTCG
TAGGCGGTTTGTCGCGTCGGCCGTGAAAACTTGGGGCTTAACCCCAAGCCTGCGGTCG
ATACGGGCAGACTTGAGTTCGGCAGGGGAGACTGGAATTCCTGGTGTAGCGGTGAAAT
GCGCAGATATCAGGAGGAACACCGGTGGCGAAGGCGGGTCTCTGGGCCGACACTGAC
GCTGAGGAGCGAAAGCGTGGGGAGCGAACAGGATTAGATACCCTGGTAGTCCACGCC
GTAAACGGTGGGTGCTAGGTGTGGGGGGCTTCCACGCCCTCTGTGCCGCAGCTAACG
CATTAAGCACCCCGCCTGGGGAGTACGGCCGCAAGGCTAAAACTCAAAGGAATTGACG
GGGGCCCGCACAAGCGGCGGAGCATGTGGATTAATTCGATGCAACGCGAAGAACCTTA
CCTGGGCTTGACATGGACTAGAAAGCTCTAGAGATAGGGCCTCCCTTGTGGCTGGTTC
ACAGGTGGTGCATGGCTGTCGTCAGCTCGTGTCGTGAGATGTTGGGTTAAGTCCCGCA
ACGAGCGCAACCCTCGTTCCATGTTGCCAGCACGTAATGGTGGGGACTCATGGGAGAC
TGCCGGGGTCAACTCGGAGGAAGGTGGGGATGACGTCAAGTCATCATGCCCCTTATGT
CCAGGGCTTCACACATGCTACAATGGCCGGTACAAAGGGCTGCTAAGCCGTGAGGTGG
AGCGAATCCCATAAAGCCGGTCTCAGTTCGGATCGGGGTCTGCAACTCGACCCCGTGA
AGTCGGAGTCGCTAGTAATCGCAGATCAGCAACGCTGCGGTGAATACGTTCCCGGGCC
TTGTACACACCGCCCGTCACGTCACGAAAGTCGGTAACACCCGAAGCCCGTGGCTCAA
CCCGCAAGGGGGAGAGCGGTCGAAGGTGGGACTGGCGATTGGGACGAAGTCGTAACA
AGTTGCTTACAGCA

Figure S1. Structures and proposed fragmentation pathways of compounds **1-6**

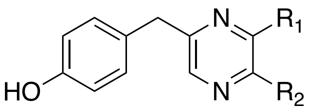

compounds **1-6**

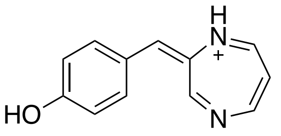

Chemical Formula: $C_{12}H_{11}N_2O^+$
Exact Mass: 199.0866

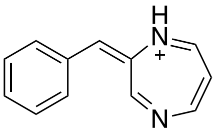

Chemical Formula: $C_{12}H_{11}N_2^+$
Exact Mass: 183.0917

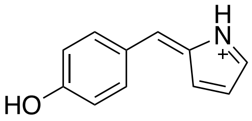

Chemical Formula: $C_{11}H_{10}NO^+$
Exact Mass: 172.0757

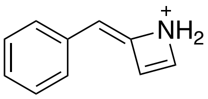

Chemical Formula: $C_{10}H_{10}N^+$
Exact Mass: 144.0808

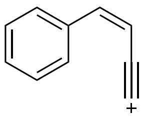

Chemical Formula: $C_{10}H_7^+$
Exact Mass: 127.0542

Figure S2. HR-ESIMS/MS spectrum of **1** in positive mode

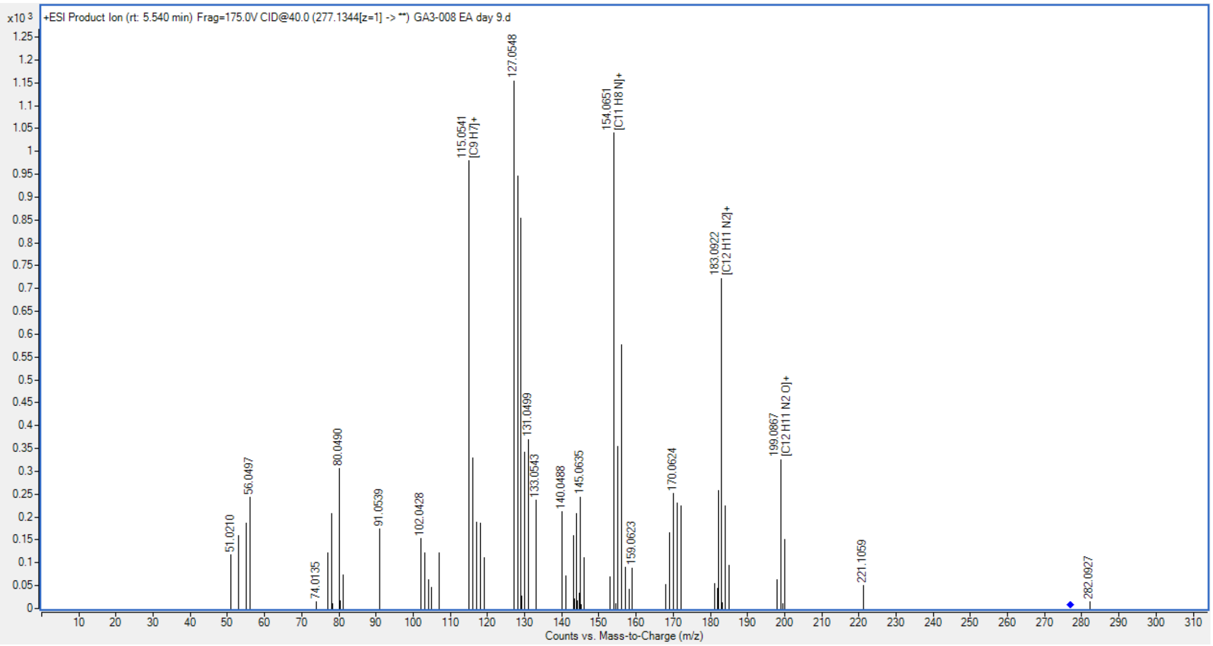

Figure S3. HR-ESIMS/MS spectrum of **2** in positive mode

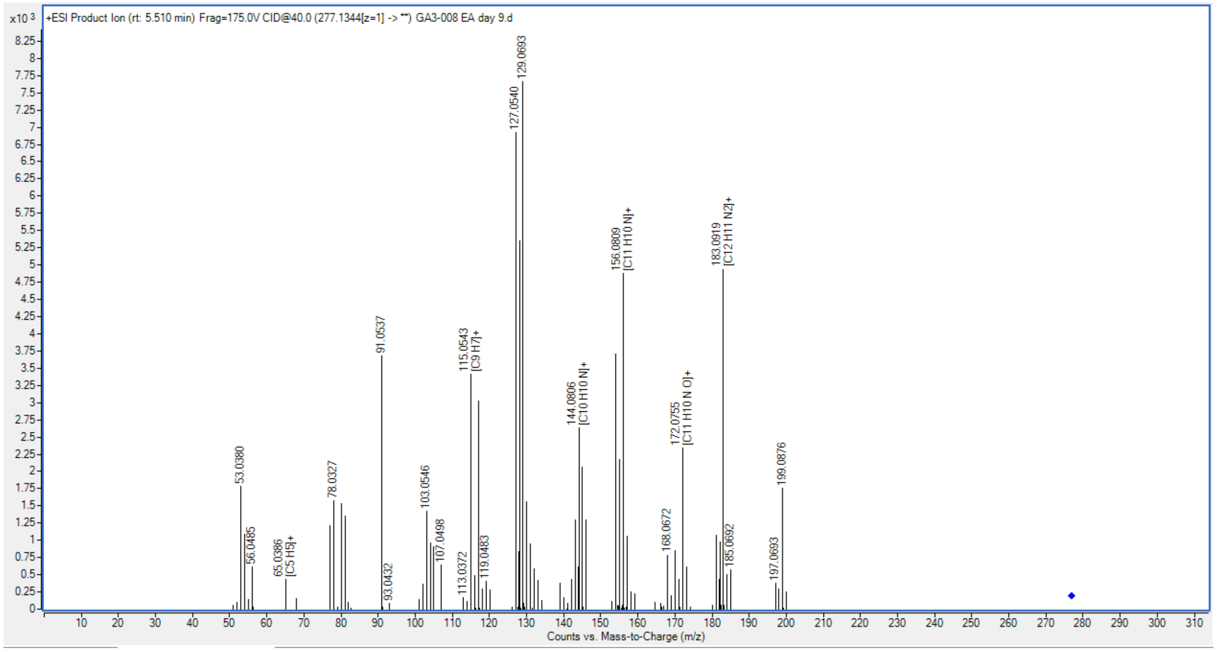

Figure S4. HR-ESIMS/MS spectrum of **3** in positive mode

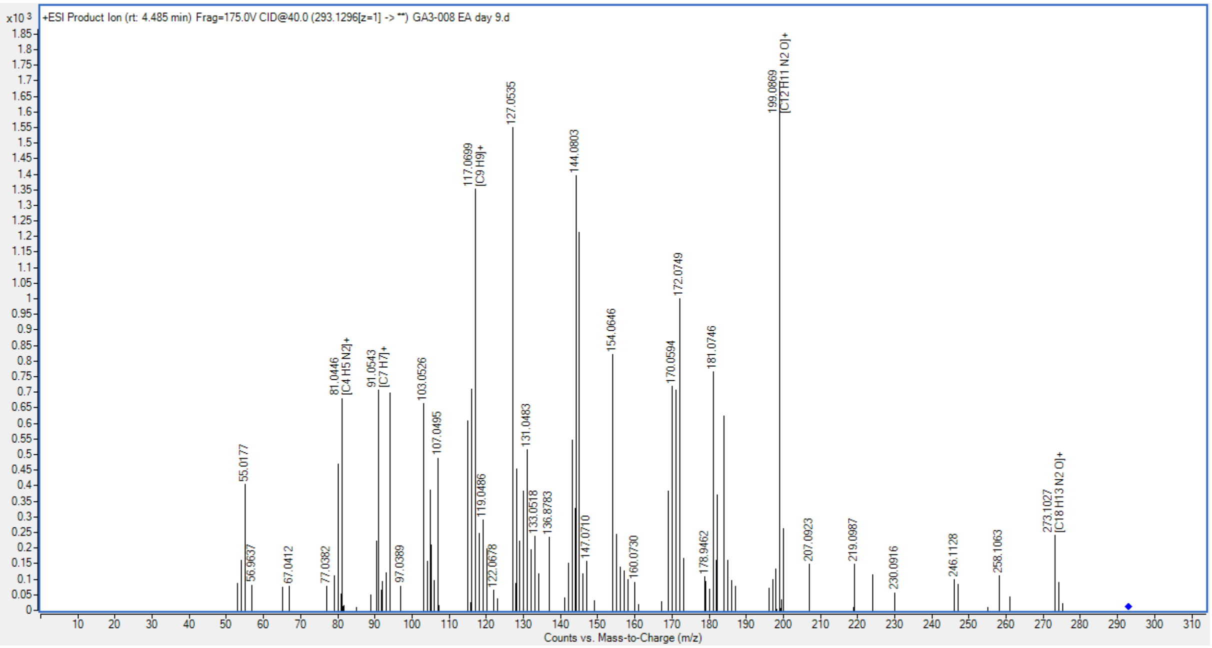

Figure S5. HR-ESIMS/MS spectrum of **4** in positive mode

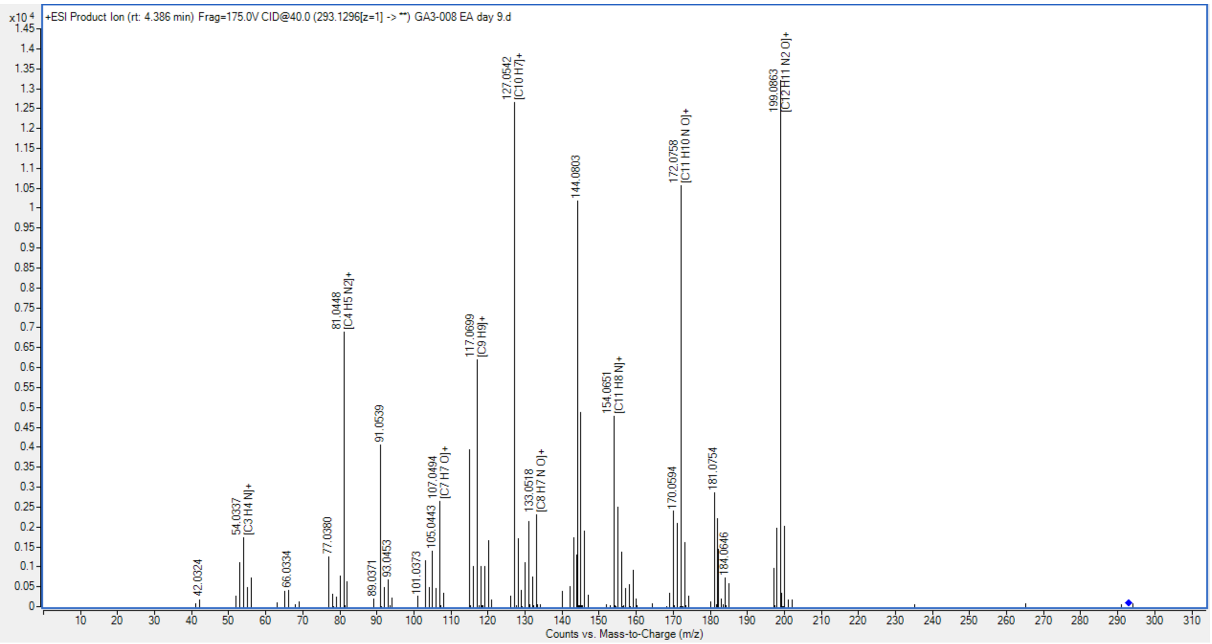

Figure S6. HR-ESIMS/MS spectrum of **5** in positive mode

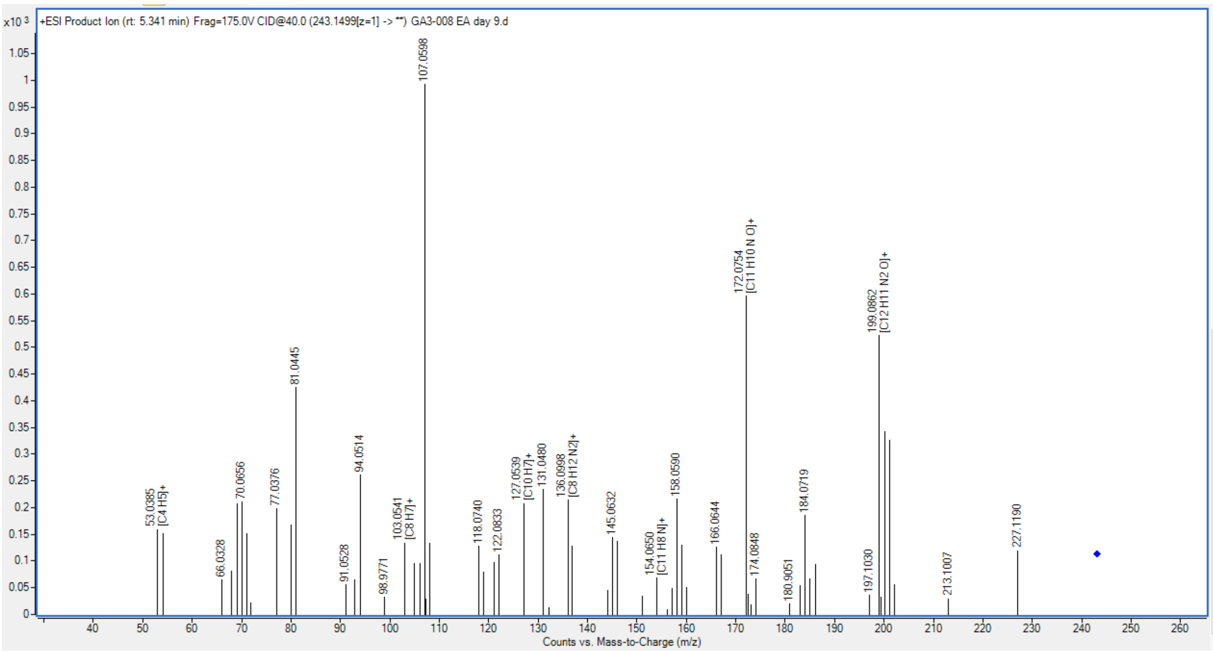

Figure S7. HR-ESIMS/MS spectrum of **6** in positive mode

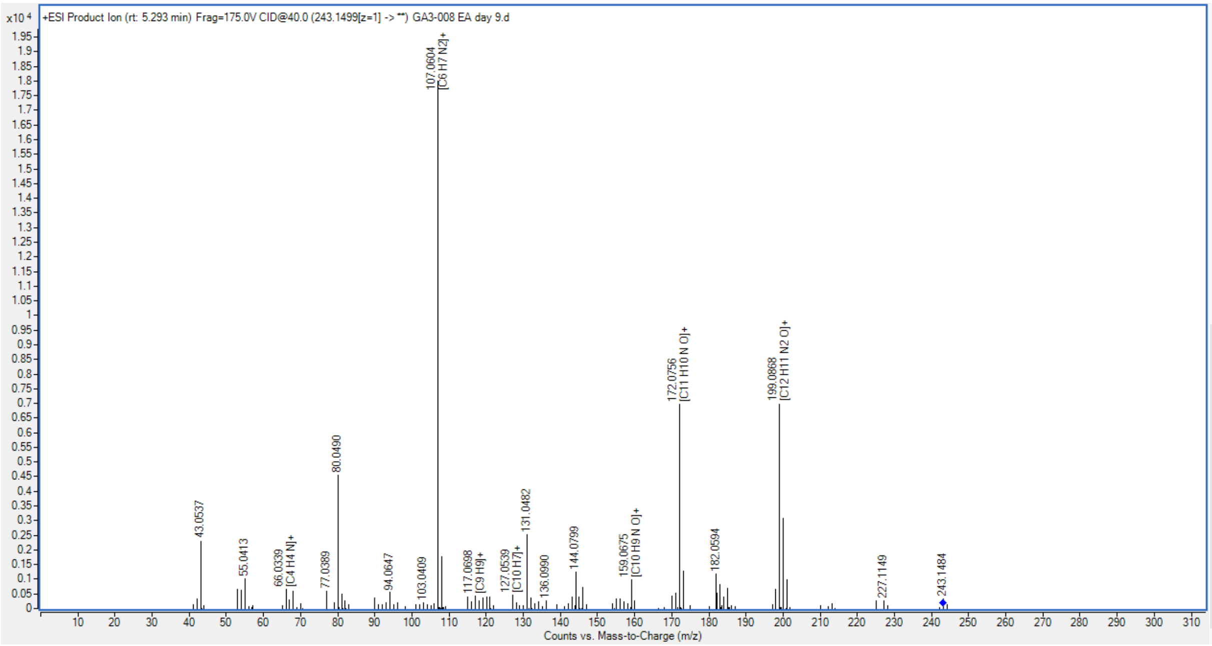

Figure S8. ^{1}H NMR spectrum of **1** in methanol-d_4 at 298 K

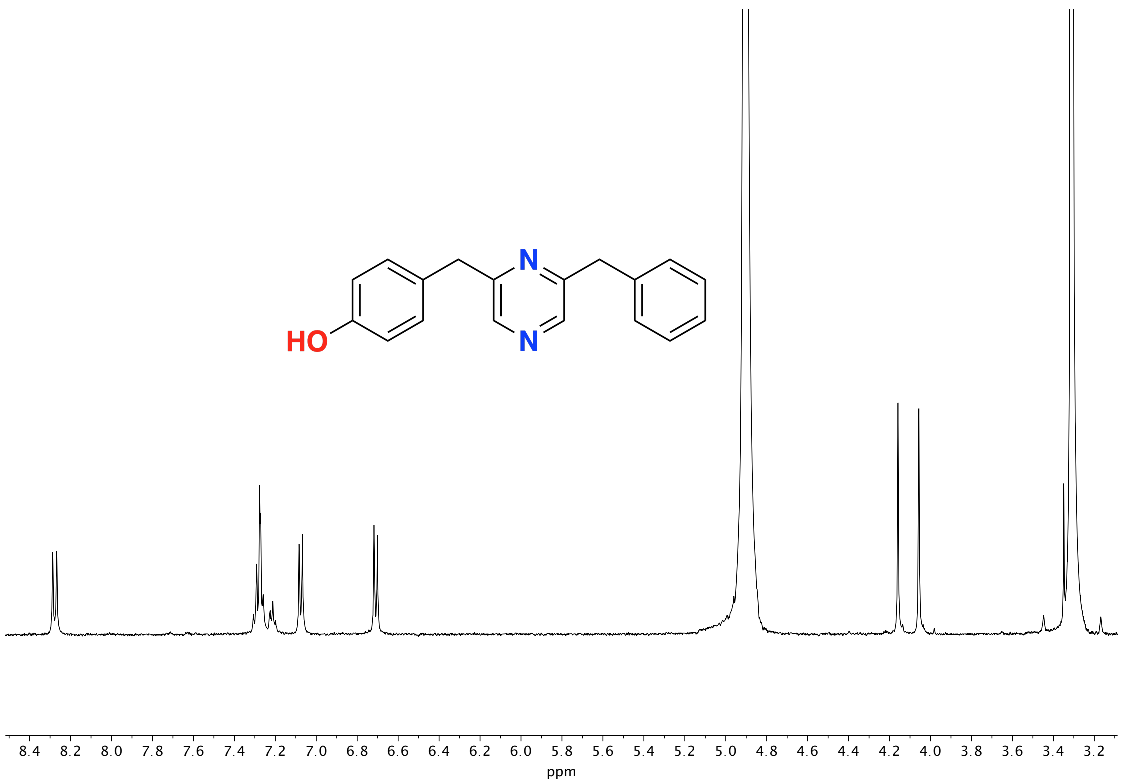

Figure S9. ^{13}C NMR spectrum of **1** in methanol-d_4 at 298 K

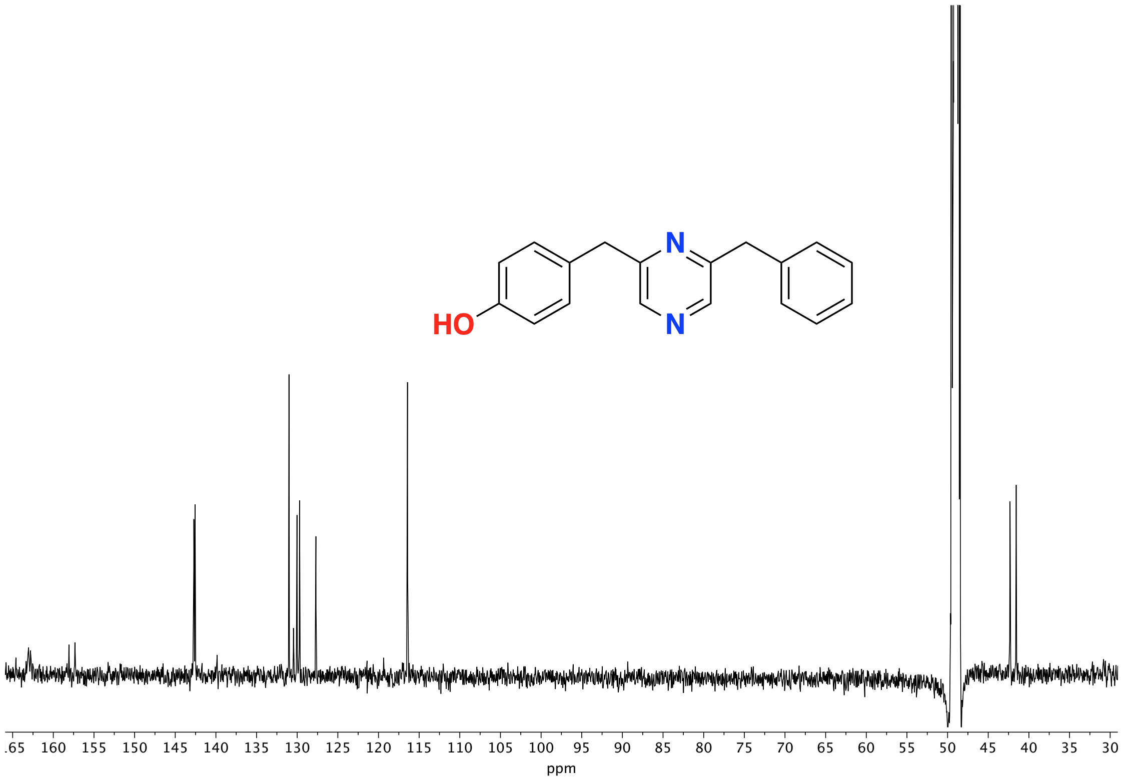

Figure S10. HSQC spectrum of **1** in methanol-d_4 at 298 K

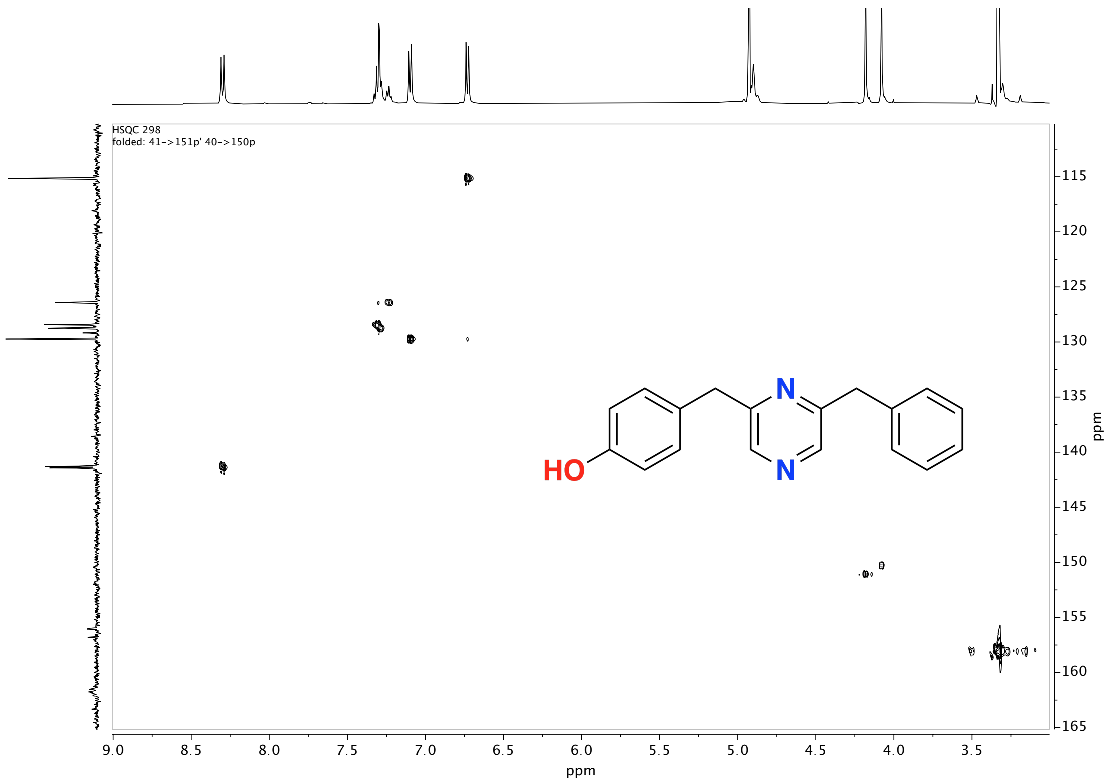

Figure S11. HMBC spectrum of **1** in methanol-d_4 at 298 K

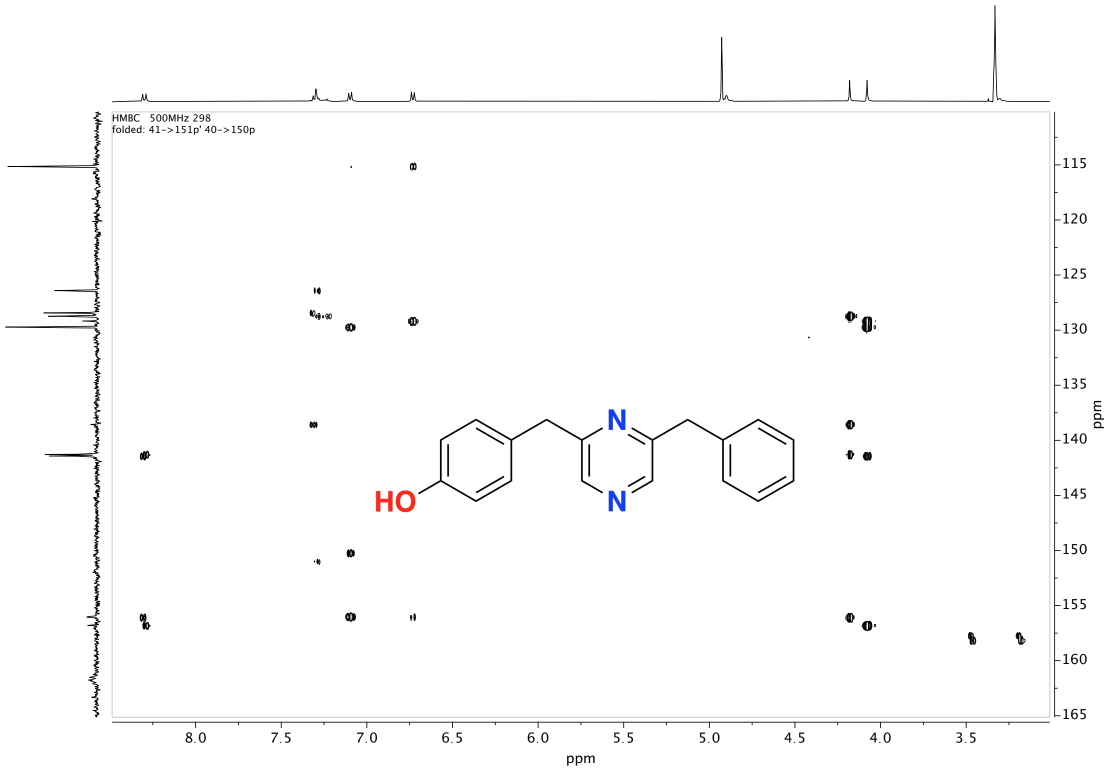

Figure S12. ^{15}N-HMBC spectrum of **1** in methanol-d_4 at 298 K

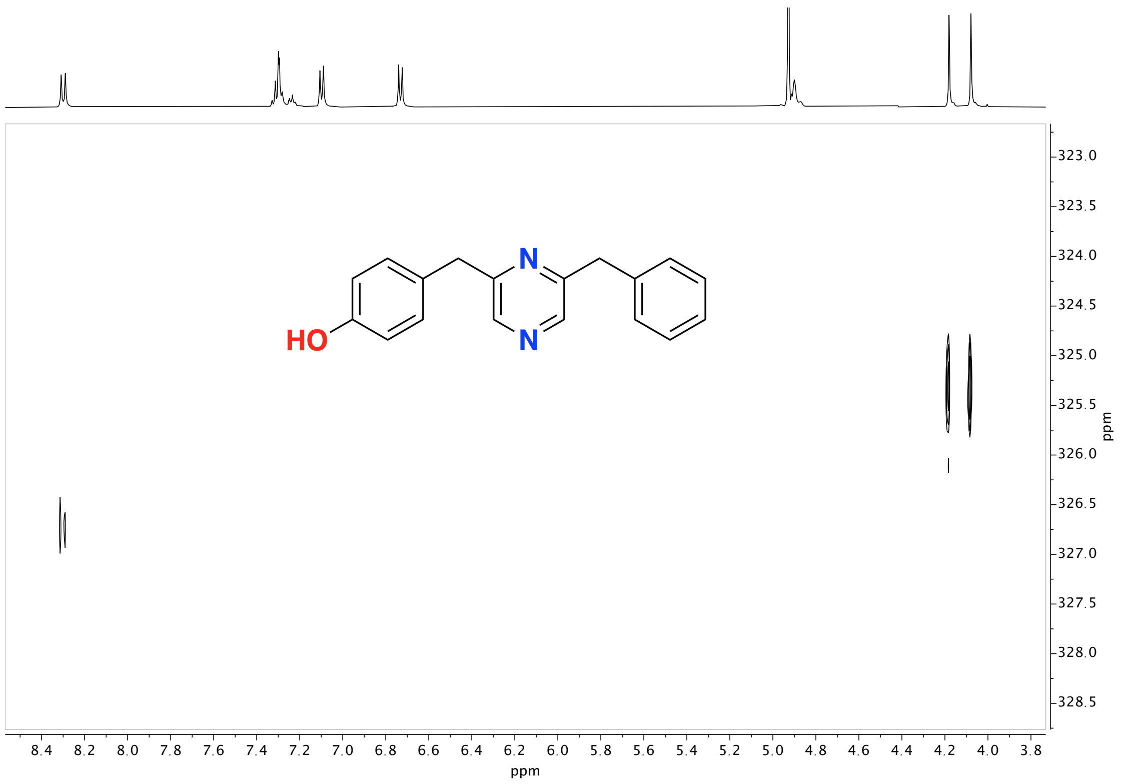

Figure S13. HRESIMS spectrum of **1**

Elemental Composition Report **Page 1**

Single Mass Analysis
Tolerance = 5.0 mDa / DBE: min = -1.5, max = 100.0
Element prediction: Off
Number of isotope peaks used for i-FIT = 3

Monoisotopic Mass, Even Electron Ions
183 formula(e) evaluated with 2 results within limits (up to 50 closest results for each mass)
Elements Used:
C: 8-20 H: 0-150 N: 0-4 O: 0-40
HBL-02JAN2019-19-4 81 (1.387) AM2 (Ar,25000.0,0.00,0.00); ABS
TOF MS ES+

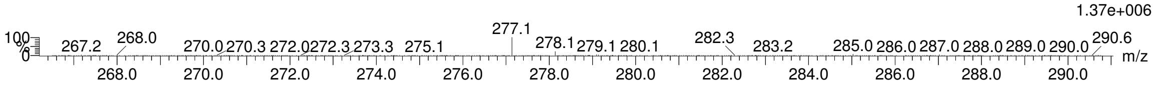

Minimum:				-1.5				
Maximum:		5.0	5.0	100.0				
Mass	Calc. Mass	mDa	PPM	DBE	i-FIT	Norm	Conf(%)	Formula
277.1345	277.1341	0.4	1.4	11.5	664.4	0.000	99.99	C18 H17 N2 O
	277.1301	4.4	15.9	7.5	673.5	9.083	0.01	C13 H17 N4 O3

Figure S14. ^{1}H NMR spectrum of **2** in methanol-d_4 at 298 K

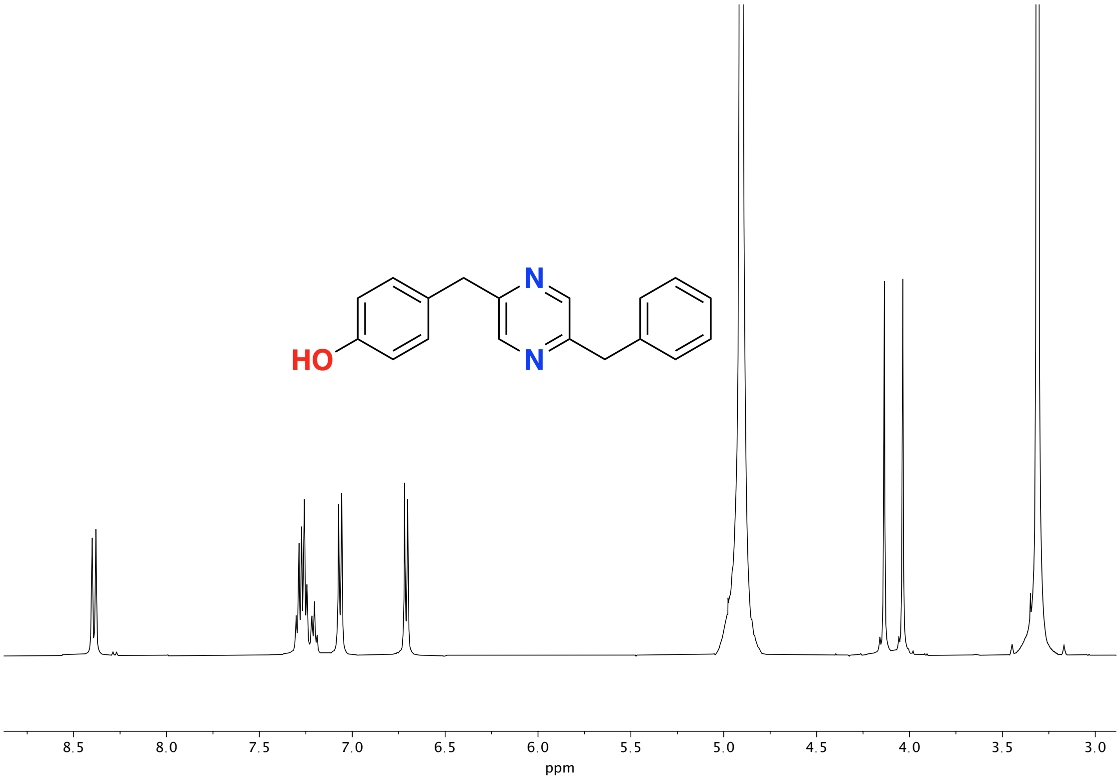

Figure S15. ^{13}C NMR spectrum of **2** in methanol-d_4 at 298 K

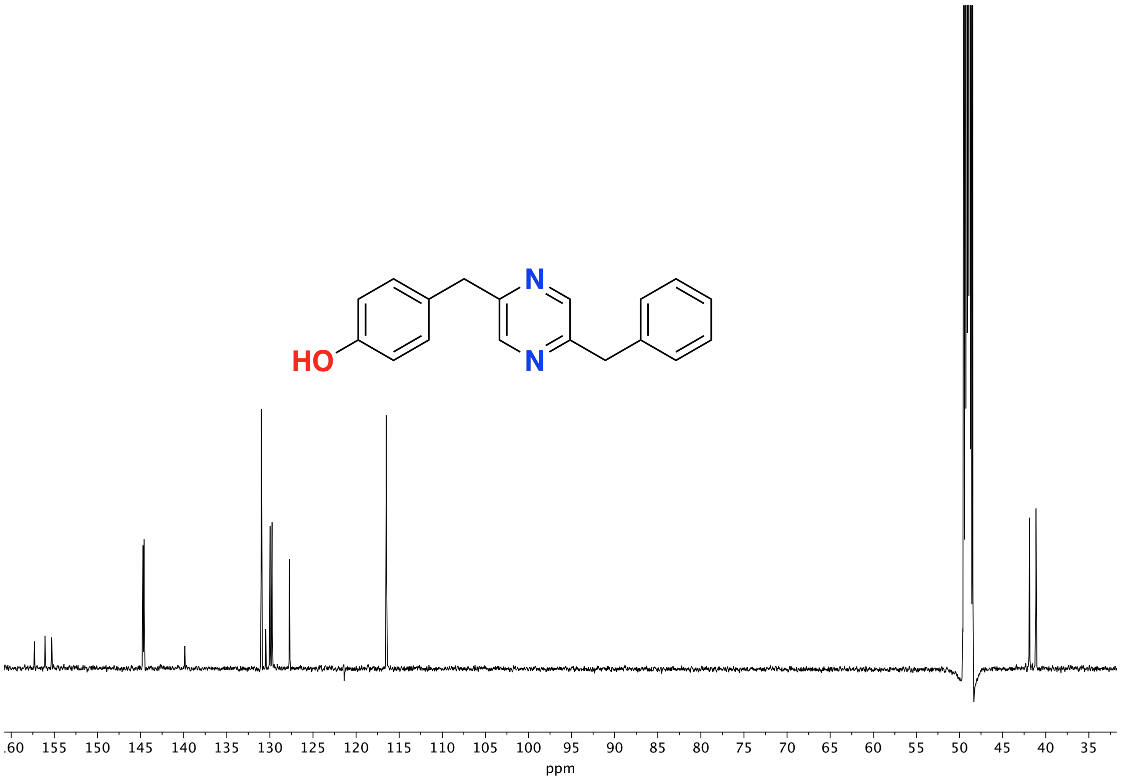

Figure S16. ^{1}H-^{1}H COSY spectrum of **2** in methanol-d_4 at 298 K

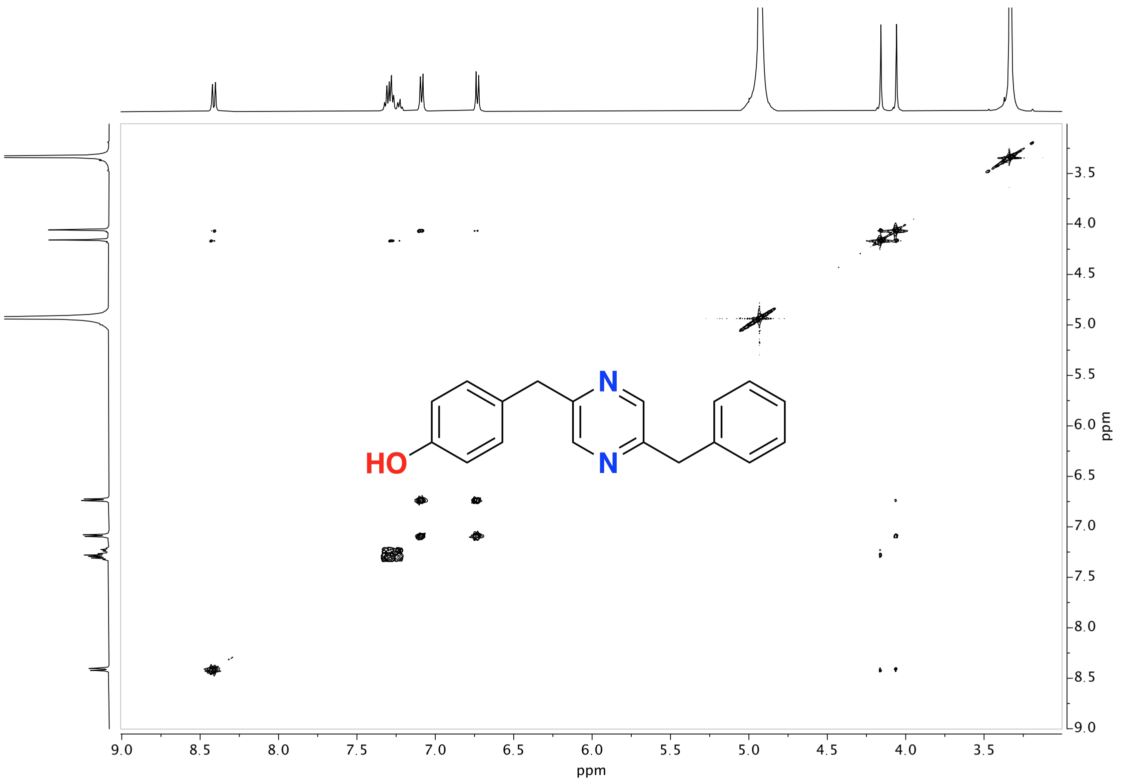

Figure S17. HSQC spectrum of **2** in methanol-d_4 at 298 K

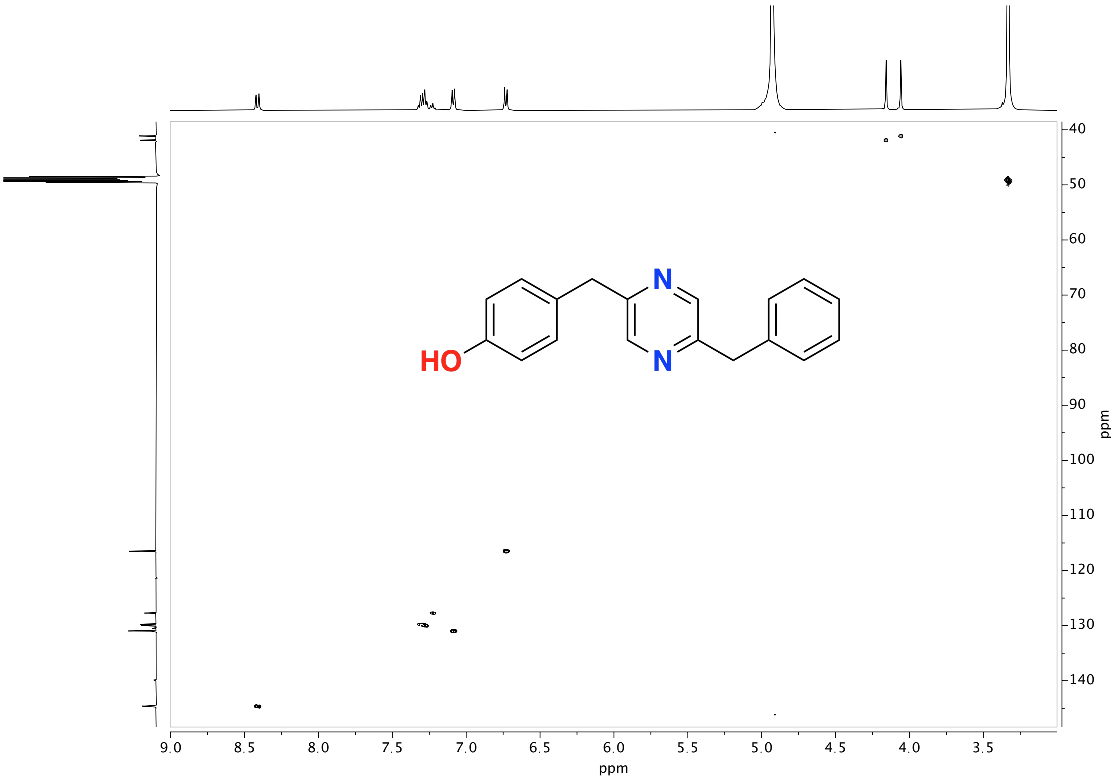

Figure S18. HMBC spectrum of **2** in methanol-d_4 at 298 K

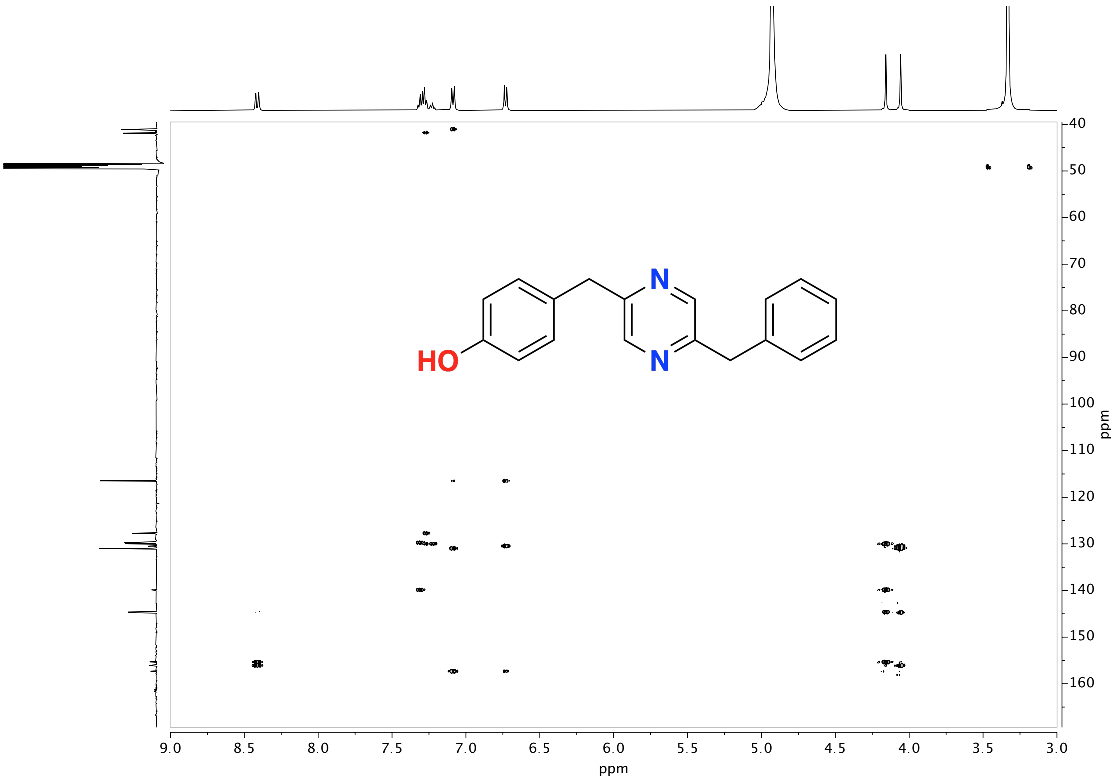

Figure S19. ^{15}N-HMBC spectrum of **2** in pyridine-d_4 at 298 K

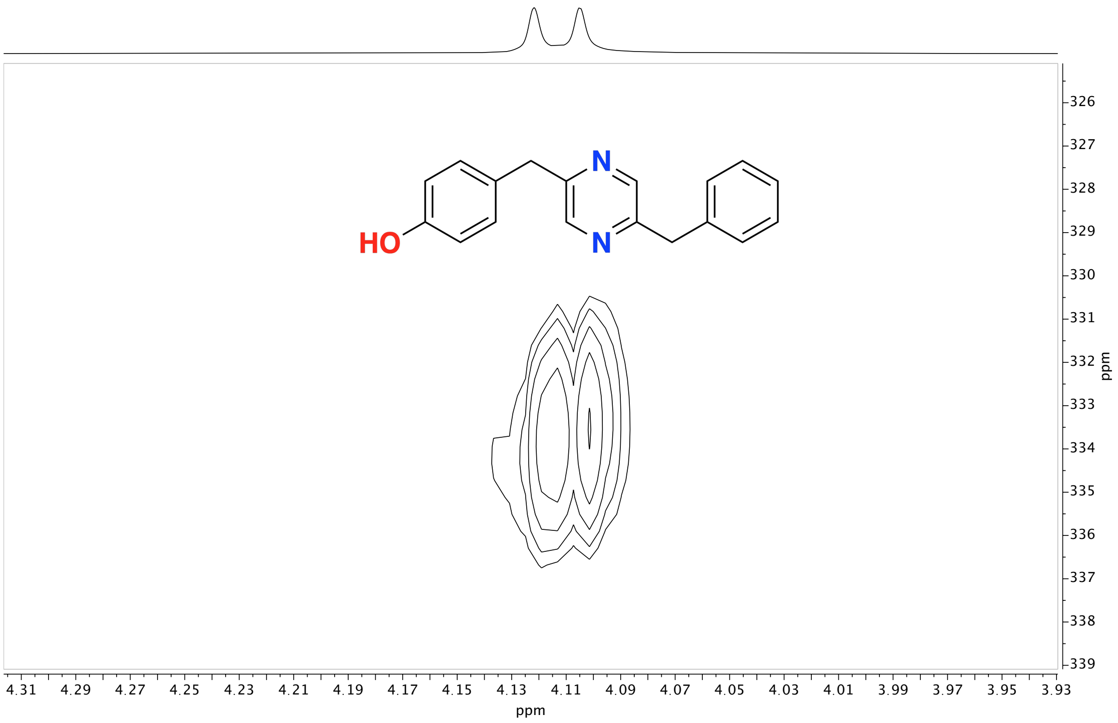

Figure S20. HRESIMS spectrum of **2**

Elemental Composition Report **Page 1**

Single Mass Analysis
Tolerance = 5.0 mDa / DBE: min = -1.5, max = 100.0
Element prediction: Off
Number of isotope peaks used for i-FIT = 3

Monoisotopic Mass, Even Electron Ions
217 formula(e) evaluated with 3 results within limits (up to 50 closest results for each mass)
Elements Used:
C: 0-30 H: 0-150 N: 0-4 O: 0-40
HBL-02JAN2019-GA3-008-19-3 209 (3.552) AM2 (Ar,25000.0,0.00,0.00); ABS
TOF MS ES+

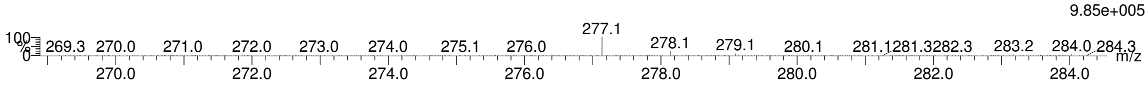

Minimum:				-1.5				
Maximum:		5.0	5.0	100.0				
Mass	Calc. Mass	mDa	PPM	DBE	i-FIT	Norm	Conf(%)	Formula
277.1343	277.1341	0.2	0.7	11.5	633.2	0.000	99.99	C18 H17 N2 O
	277.1359	-1.6	-5.8	-1.5	646.7	13.538	0.00	C6 H21 N4 O8
	277.1301	4.2	15.2	7.5	642.4	9.191	0.01	C13 H17 N4 O3

Figure S21. ^{1}H NMR spectrum of **3** in DMSO-d_6 at 298 K

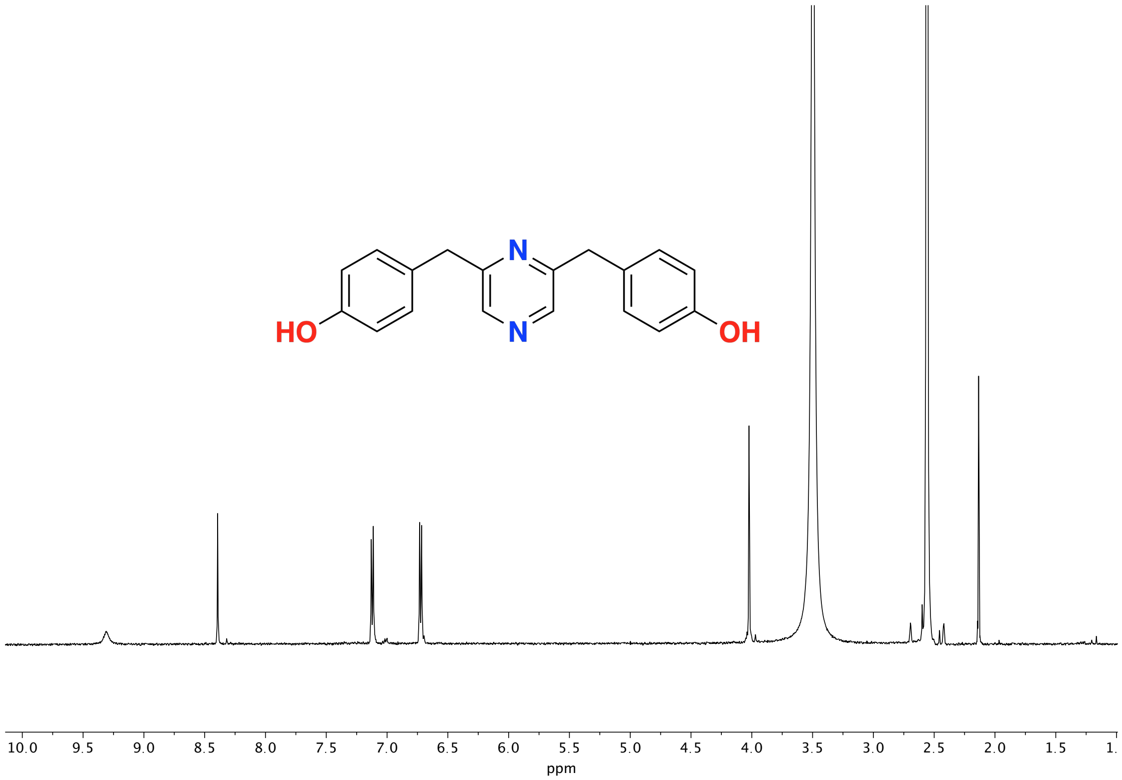

Figure S22. ^{13}C NMR spectrum of **3** in DMSO-d_6 at 298 K

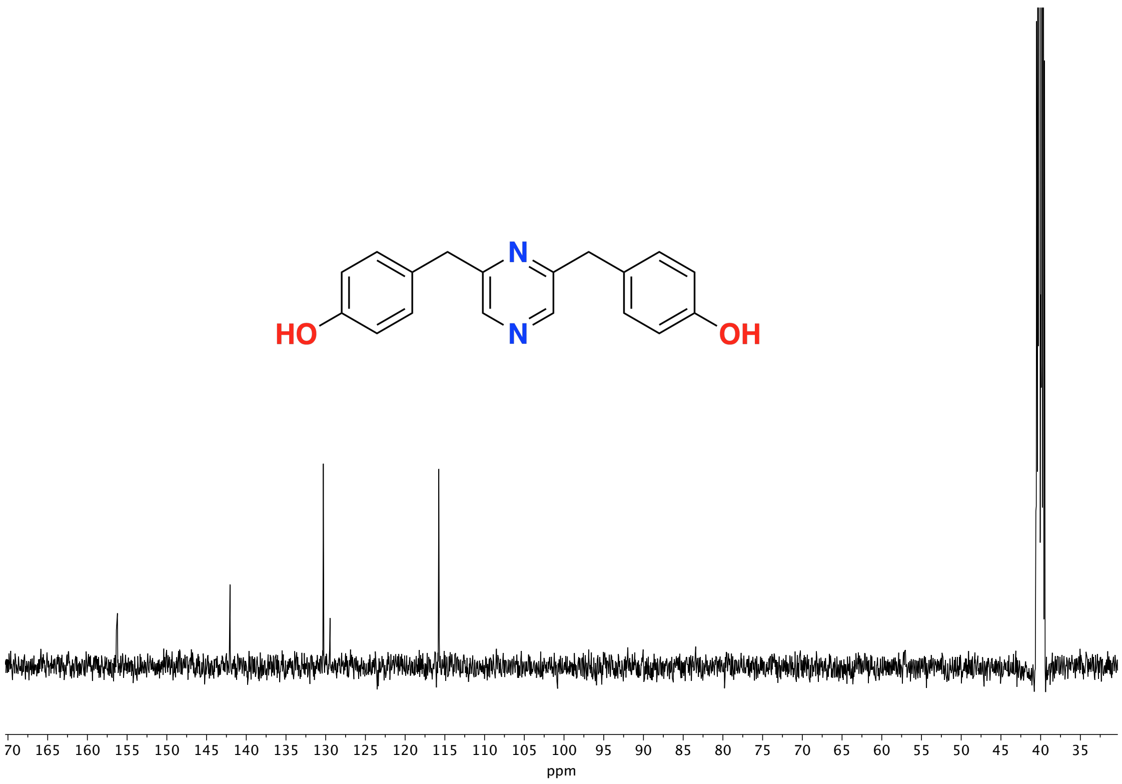

Figure S23. HSQC spectrum of **3** in DMSO-d_6 at 298 K

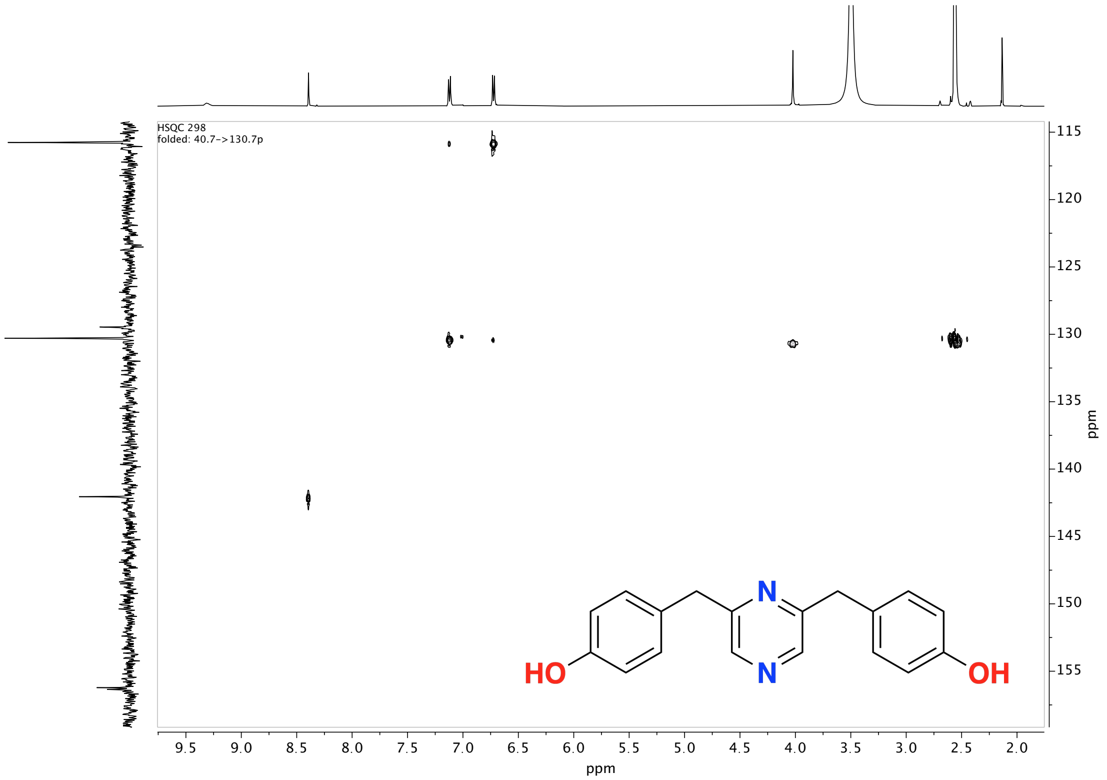

Figure S24. HMBC spectrum of **3** in DMSO-d_6 at 298 K

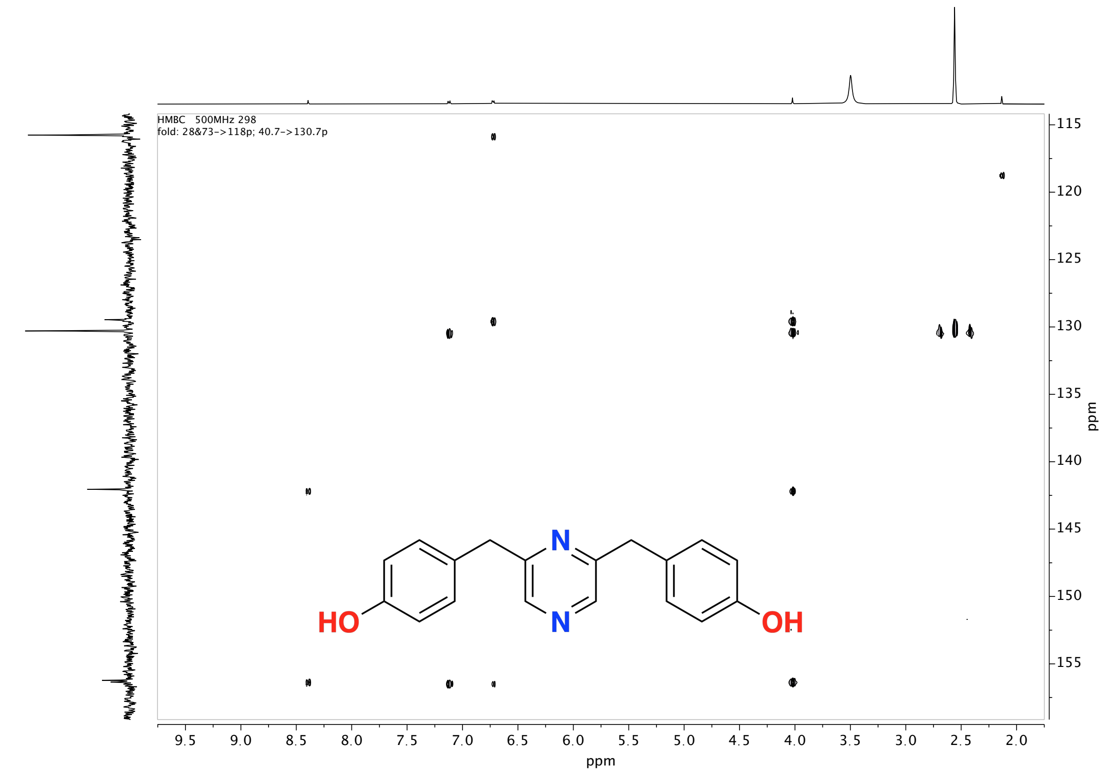

Figure S25. ^{15}N-HMBC spectrum of **3** in DMSO-d_6 at 298 K

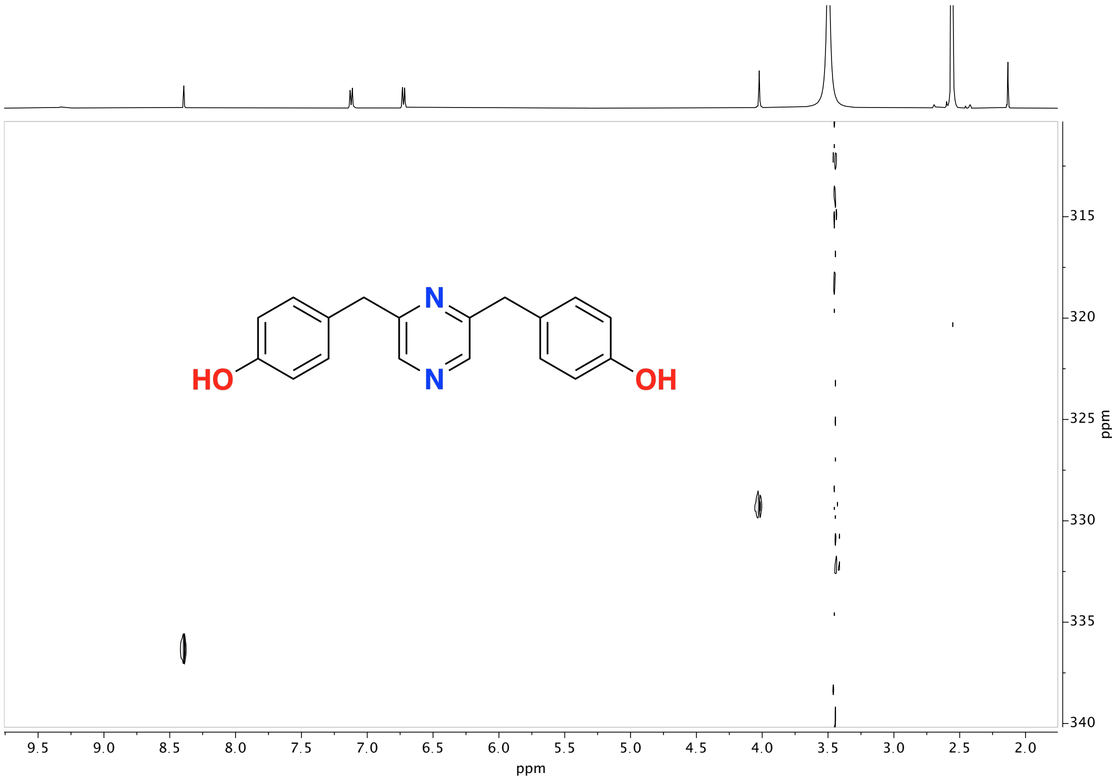

Figure S26. HRESIMS spectrum of **3**

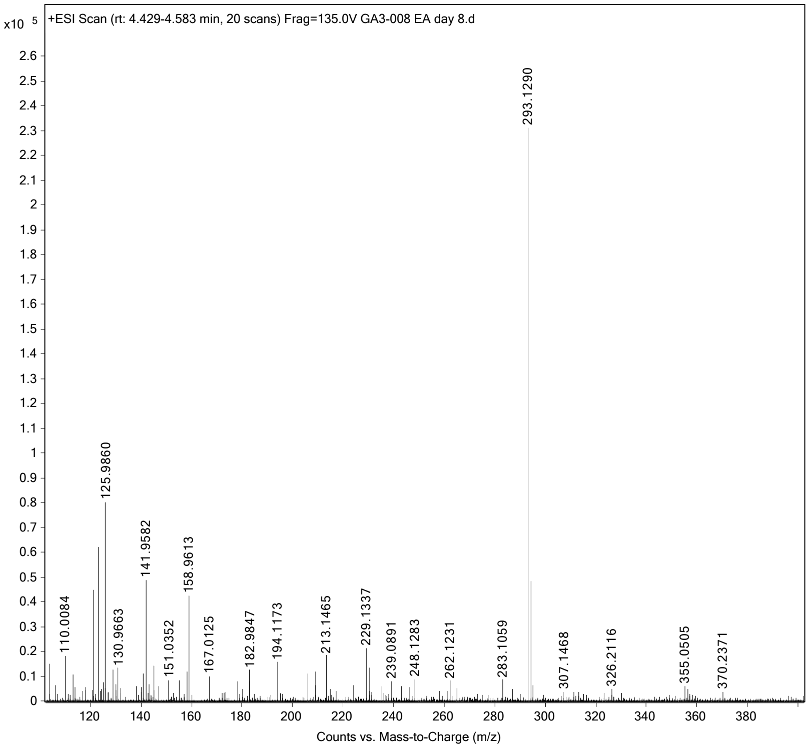

Figure S27. ^{1}H NMR spectrum of **4** in DMSO-d_6 at 298 K

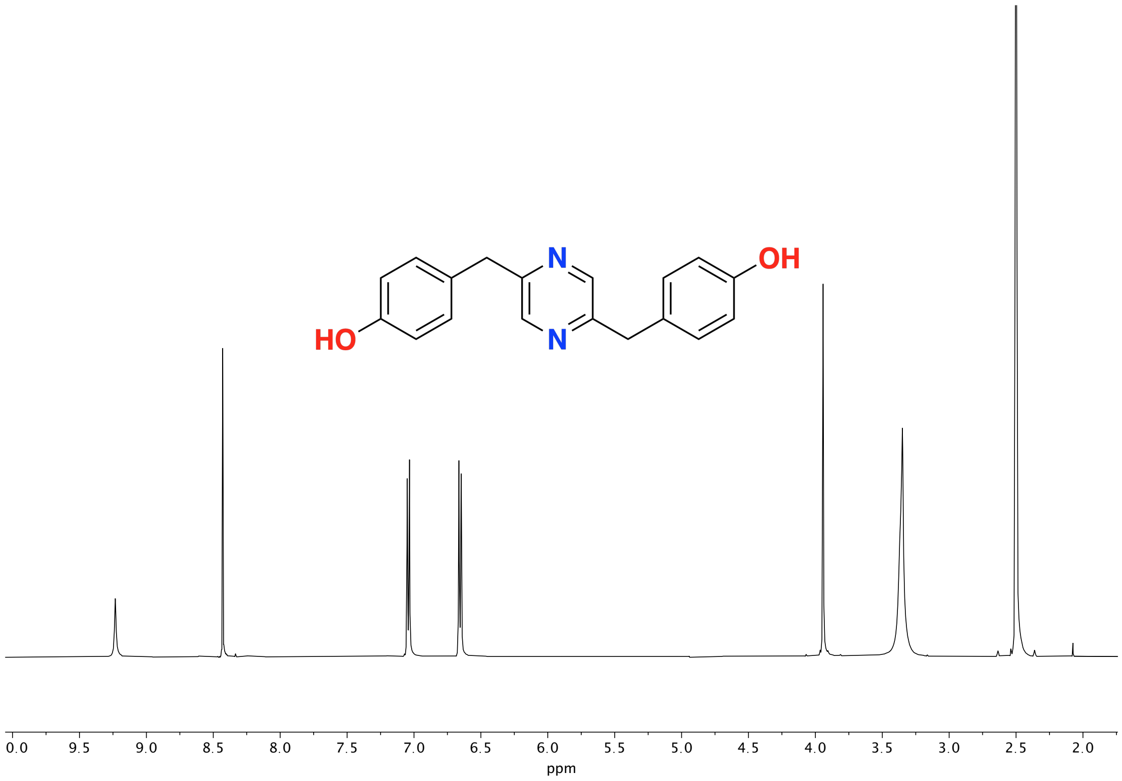

Figure S28. ^{13}C NMR spectrum of **4** in DMSO-d_6 at 298 K

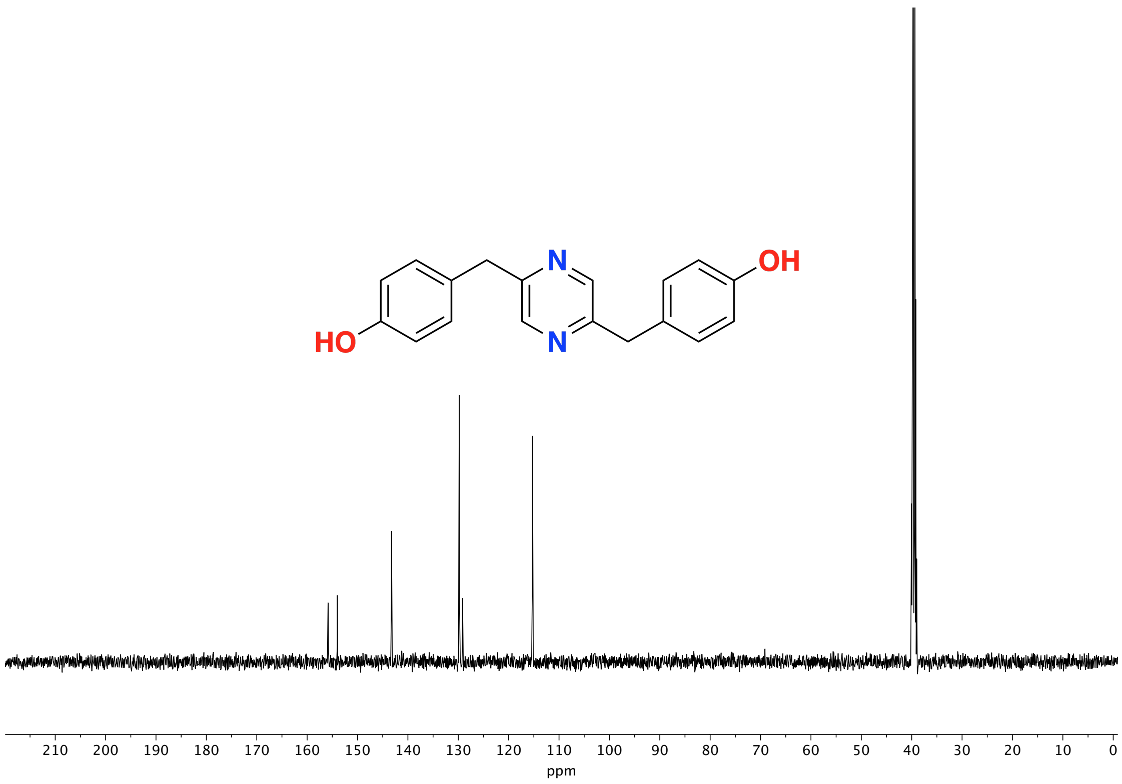

Figure S29. HMBC spectrum of **4** in DMSO-d_6 at 298 K

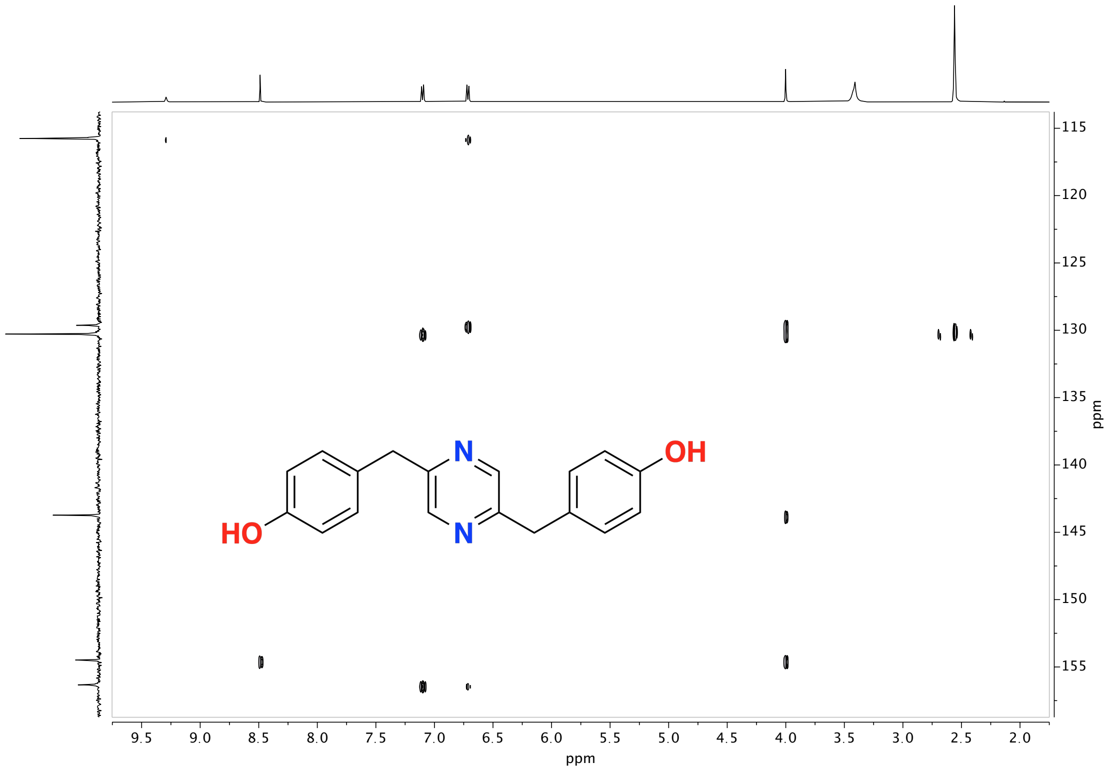

Figure S30. ^{15}N-HMBC spectrum of **4** in DMSO-d_6 at 298 K

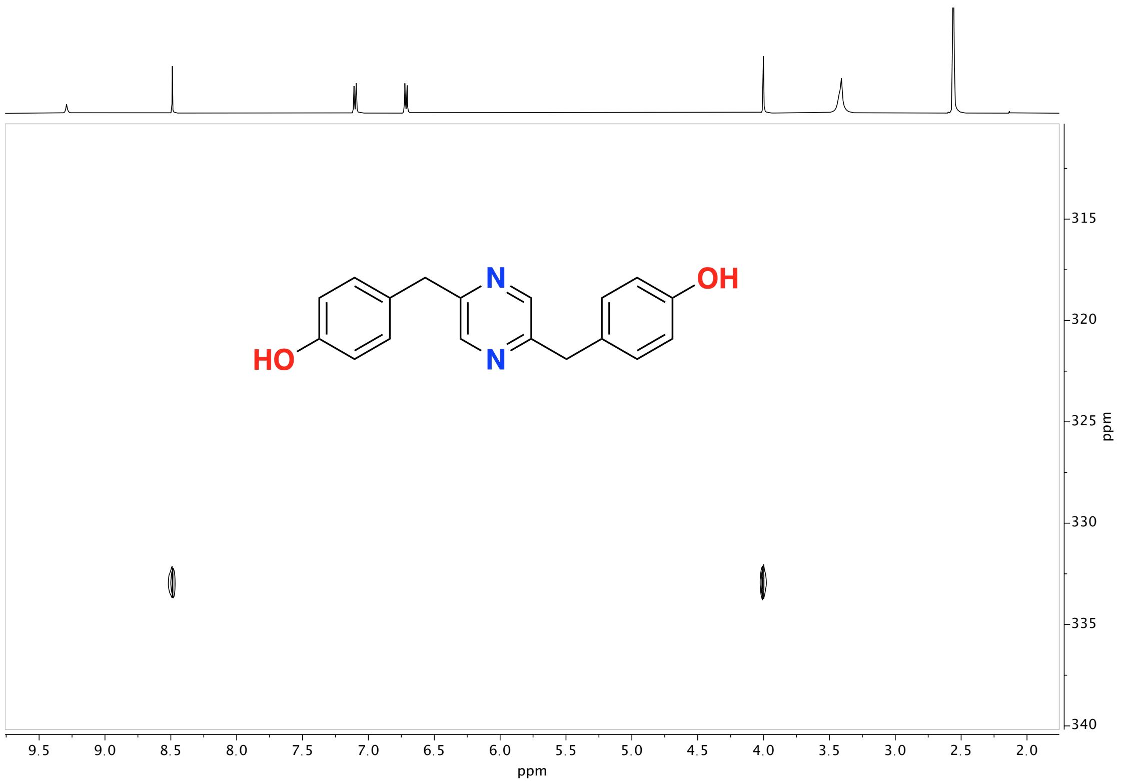

Figure S31. HRESIMS spectrum of **4**

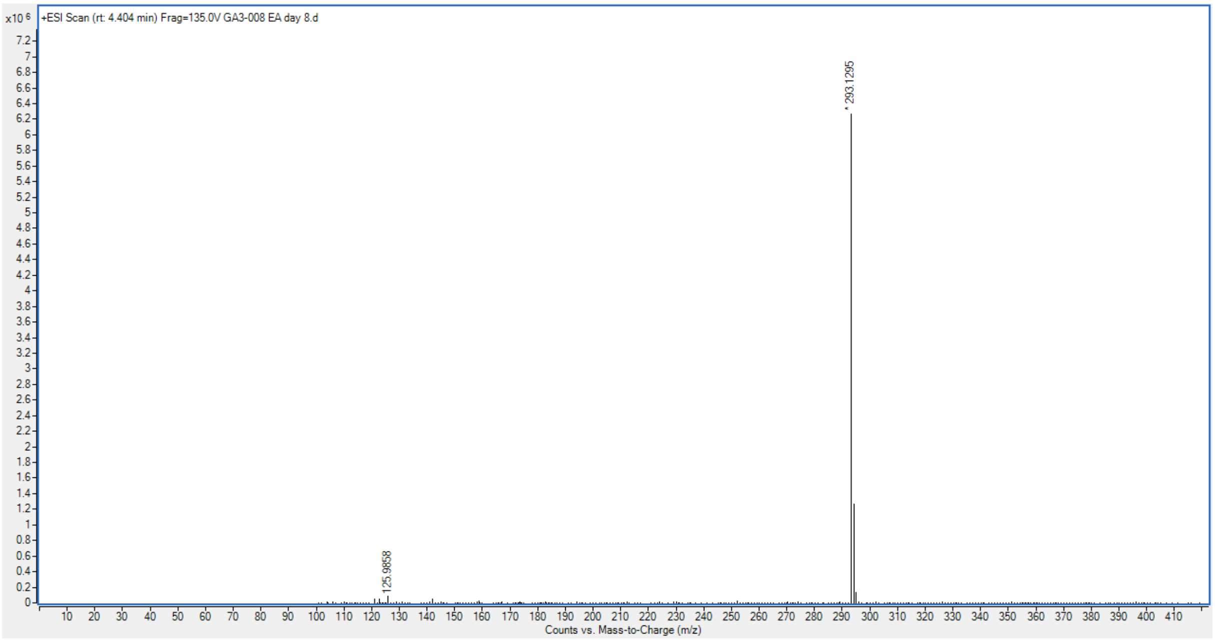

Figure S32. ^{1}H NMR spectrum of **5** in methanol-d_4 at 298 K

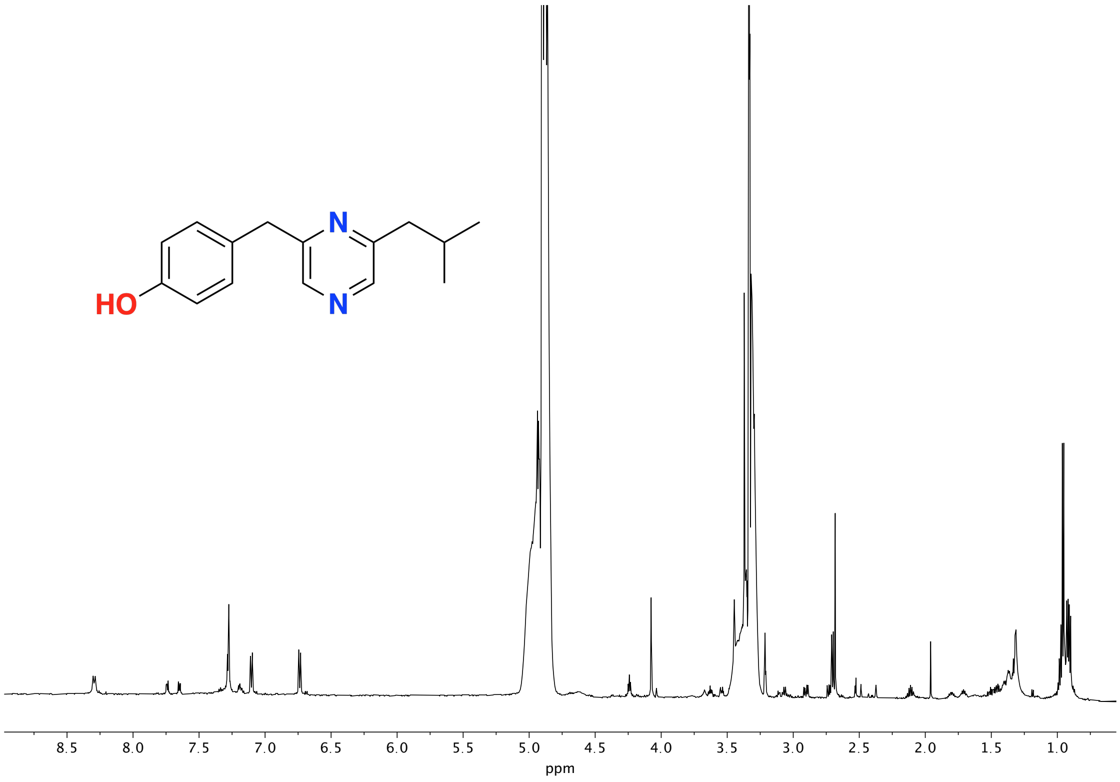

Figure S33. ${}^{13}C$ NMR spectrum of **5** in methanol-d_4 at 298 K

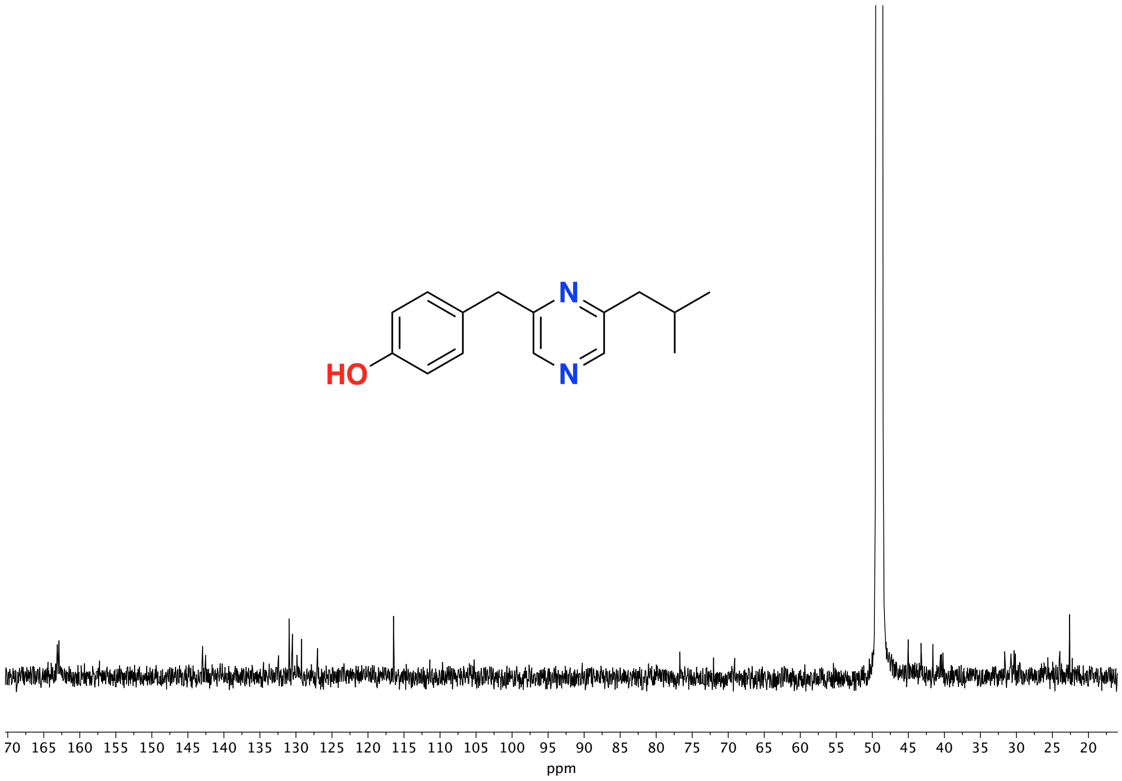

Figure S34. ${}^{1}H$-${}^{1}H$ COSY spectrum of **5** in methanol-d_4 at 298 K

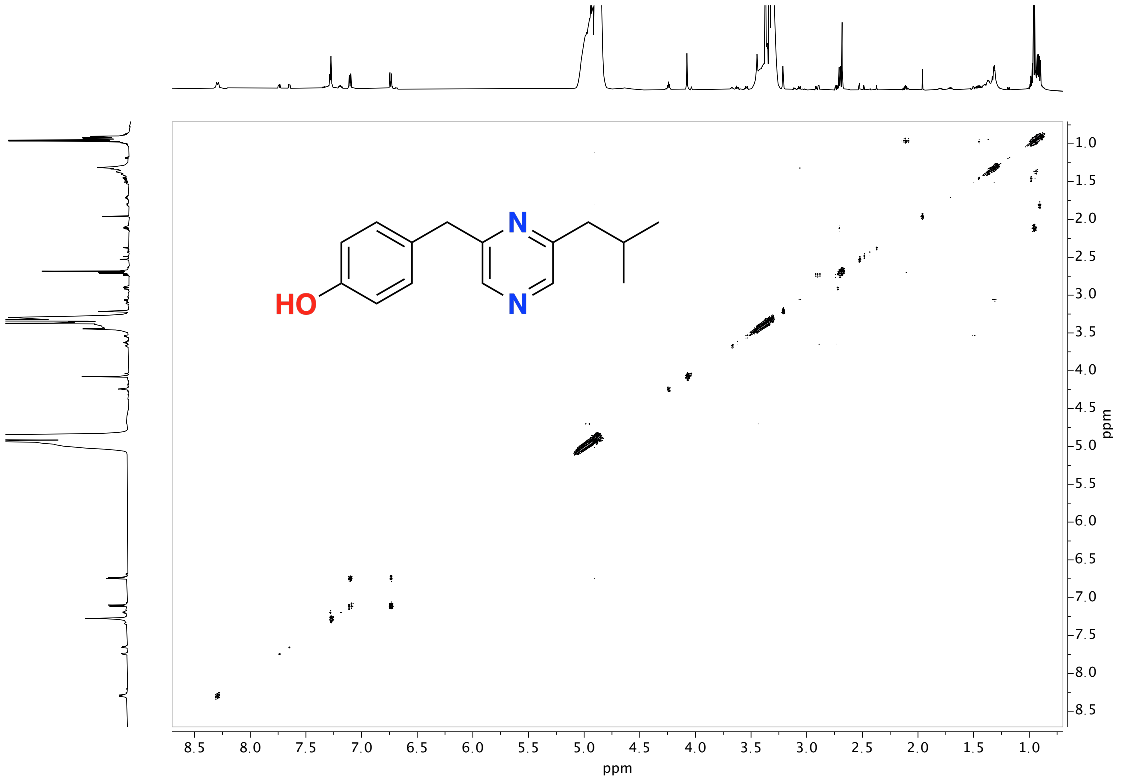

Figure S35. HSQC spectrum of **5** in methanol-d_4 at 298 K

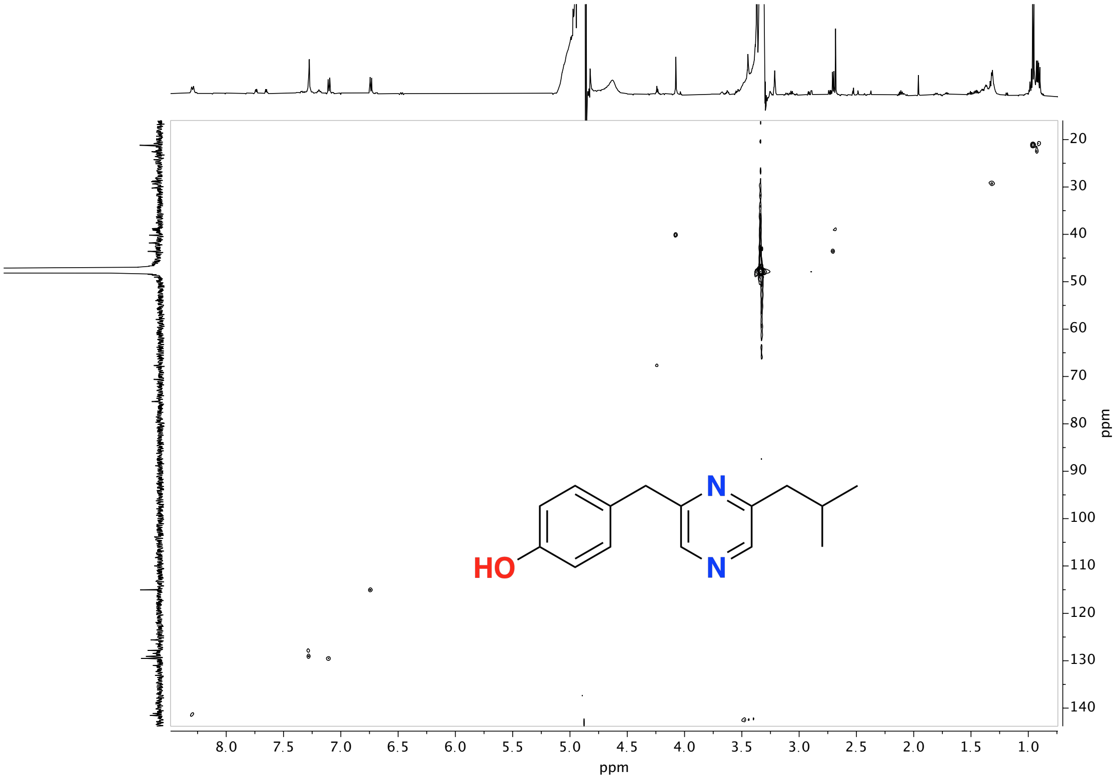

Figure S36. HMBC spectrum of **5** in methanol-d_4 at 298 K

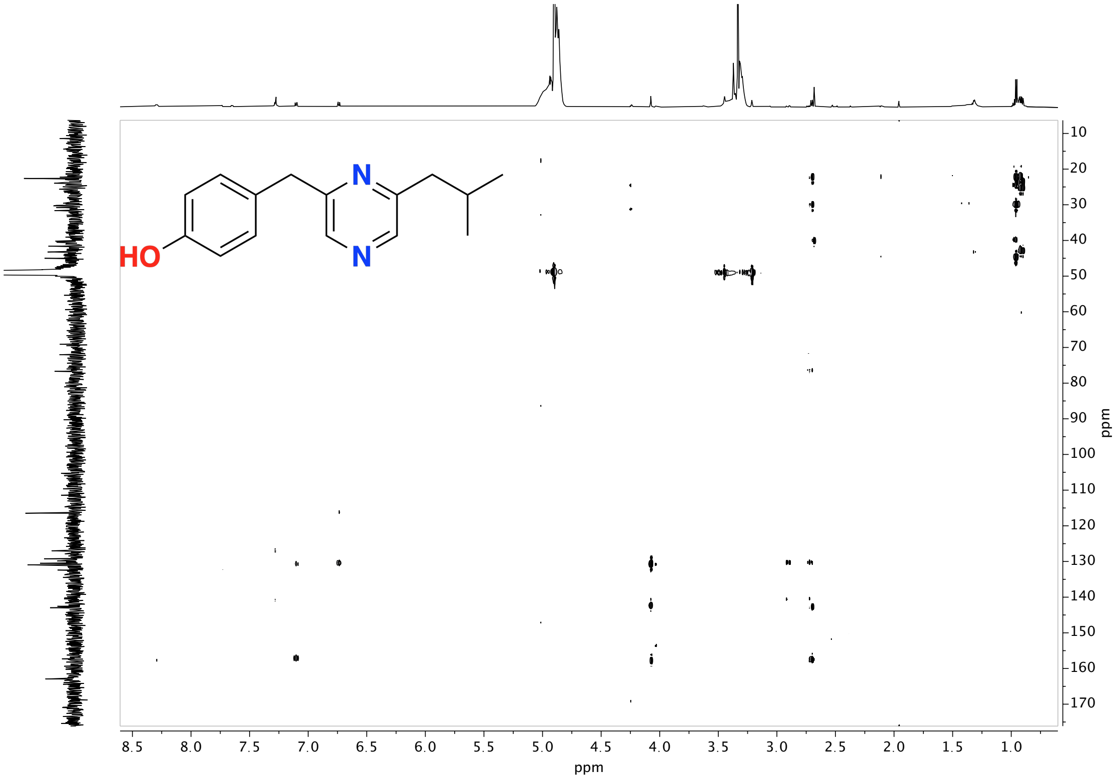

Figure S37. HRESI-MS spectrum of **5**

Elemental Composition Report **Page 1**

Single Mass Analysis
Tolerance = 5.0 mDa / DBE: min = -1.5, max = 100.0
Element prediction: Off
Number of isotope peaks used for i-FIT = 3

Monoisotopic Mass, Even Electron Ions
147 formula(e) evaluated with 2 results within limits (up to 50 closest results for each mass)
Elements Used:
C: 8-20 H: 0-150 N: 0-4 O: 0-40
HBL-02JAN2019-19-2 87 (1.488) AM2 (Ar,25000.0,0.00,0.00); ABS
TOF MS ES+

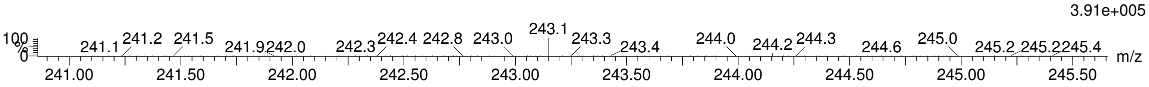

Mass	Calc. Mass	mDa	PPM	DBE	i-FIT	Norm	Conf(%)	Formula
Minimum:				-1.5				
Maximum:		5.0	5.0	100.0				
243.1497	243.1497	0.0	0.0	7.5	580.0	0.801	44.91	C15 H19 N2 O
	243.1457	4.0	16.5	3.5	579.8	0.596	55.09	C10 H19 N4 O3

Figure S38. ^{1}H NMR spectrum of **6** in methanol-d_4 at 298 K

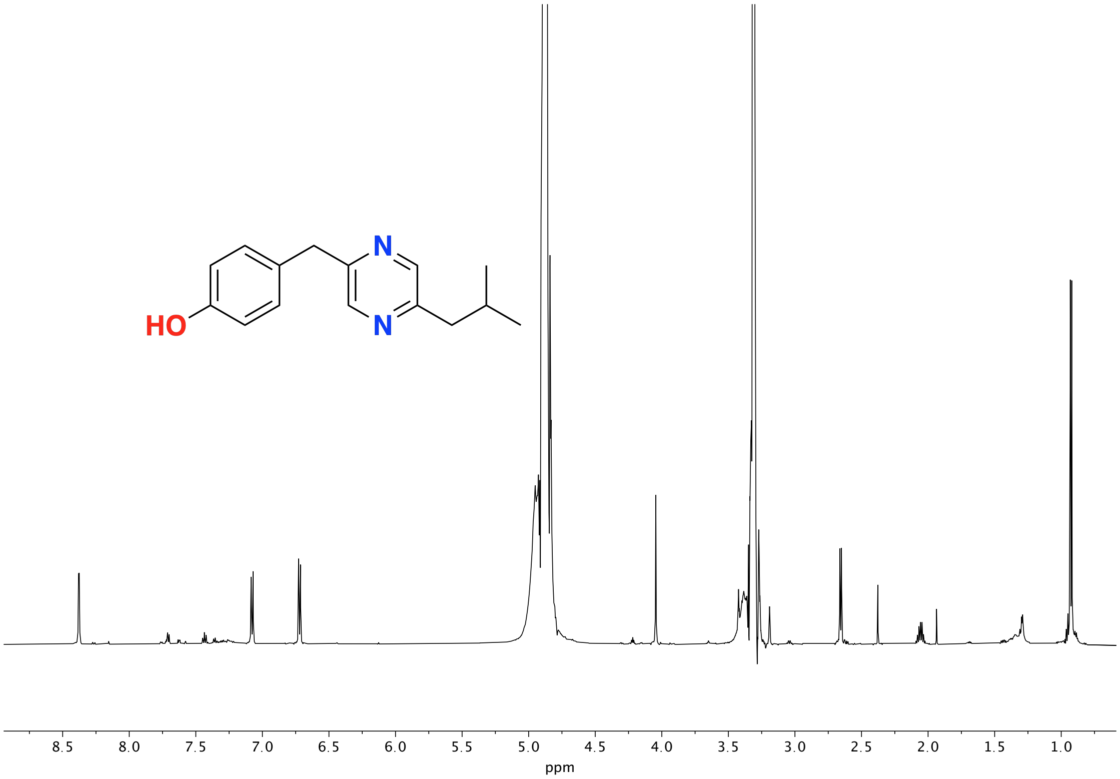

Figure S39. ^{13}C NMR spectrum of **6** in methanol-d_4 at 298 K

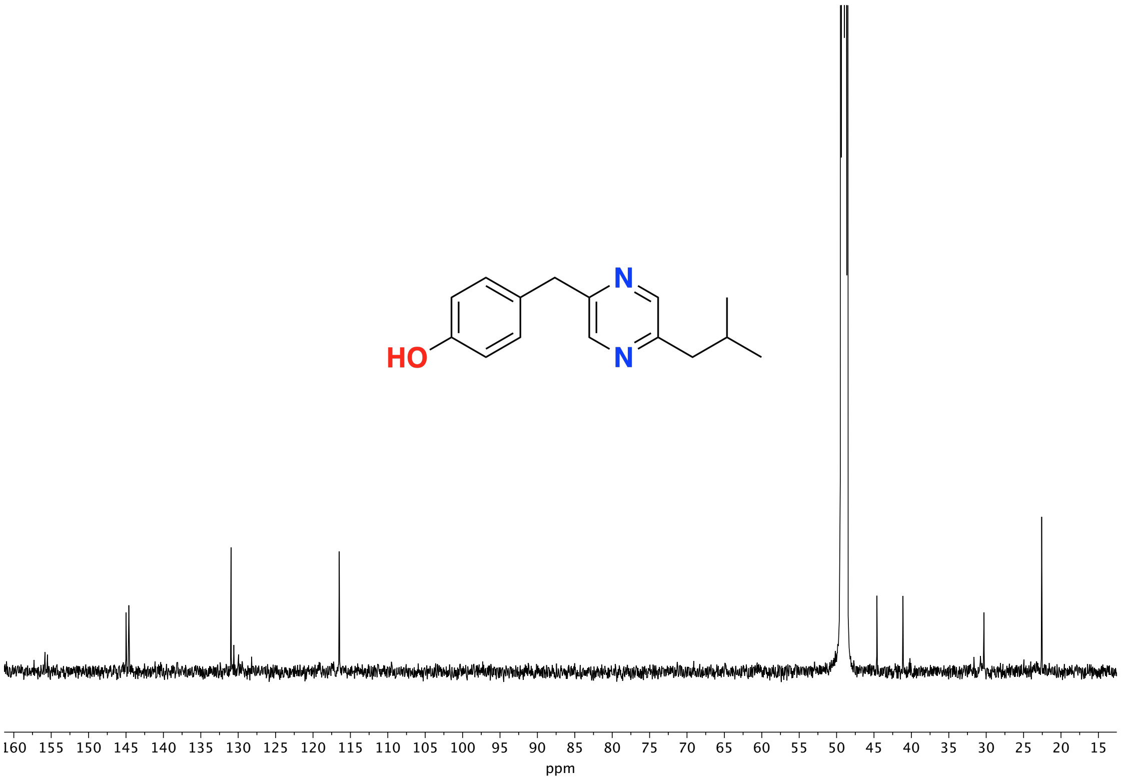

Figure S40. ^{1}H-^{1}H COSY spectrum of **6** in methanol-d_4 at 298 K

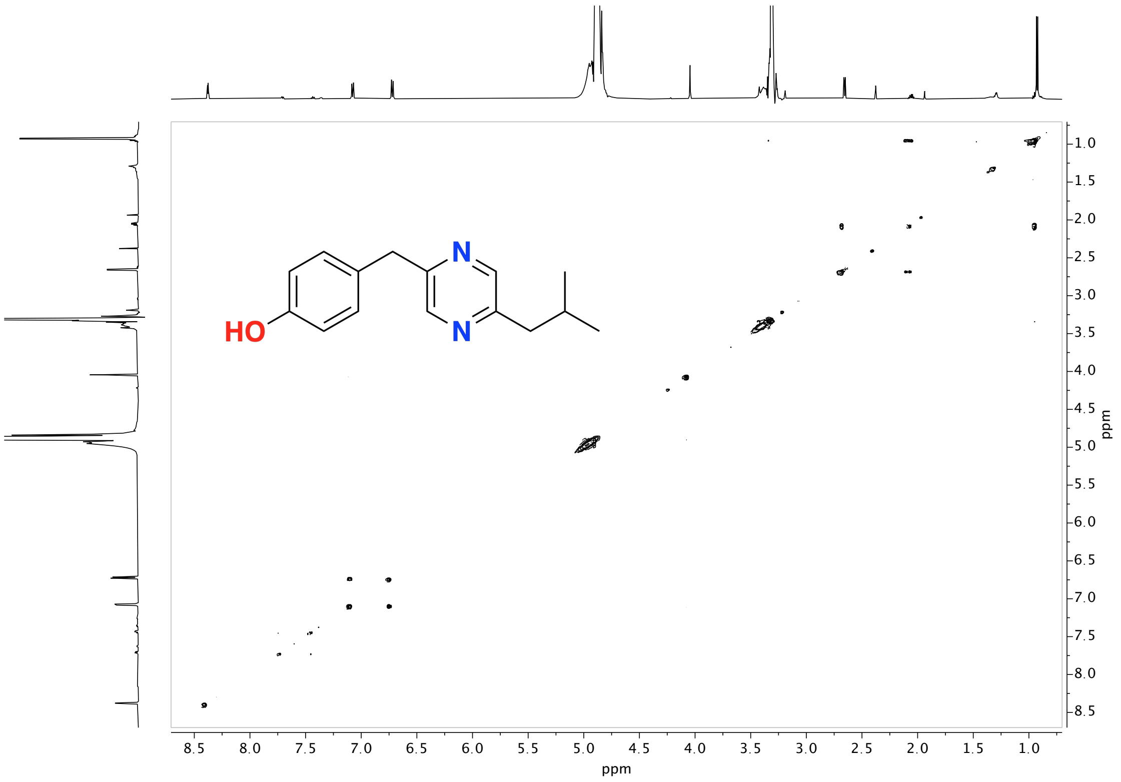

Figure S41. HSQC spectrum of **6** in methanol-d_4 at 298 K

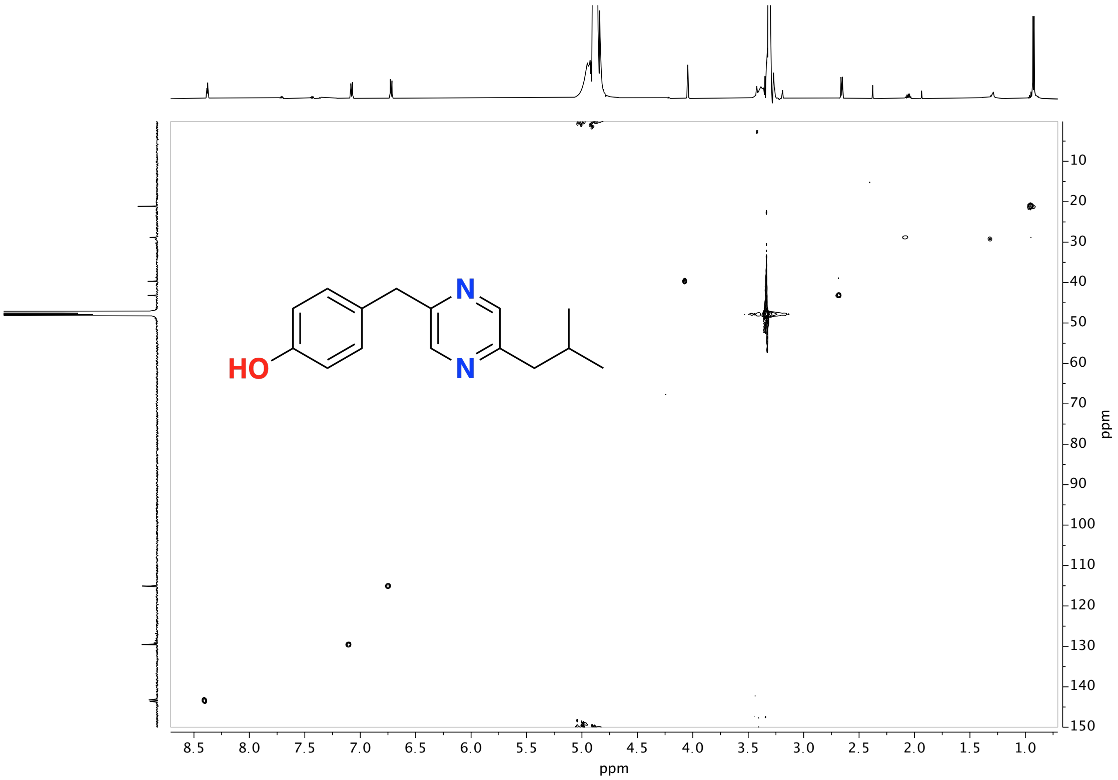

Figure S42. HMBC spectrum of **6** in methanol-d_4 at 298 K

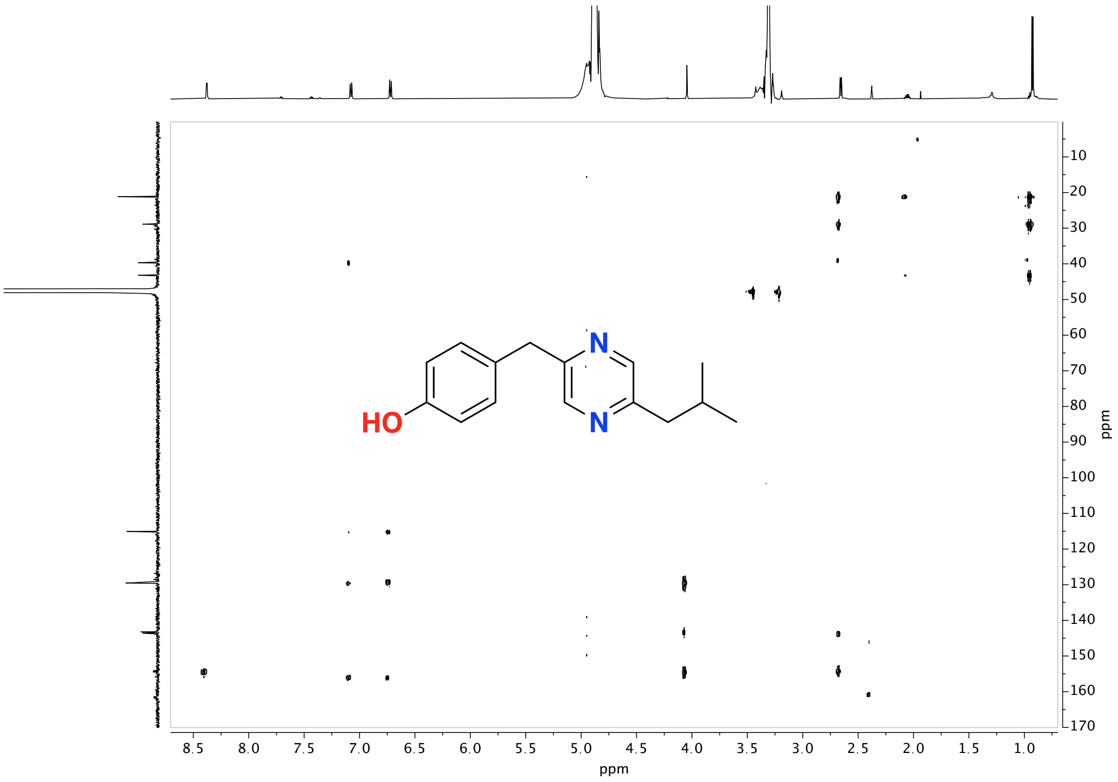

Figure S43. ^{15}N-HMBC spectrum of **6** in methanol-d_4 at 298 K

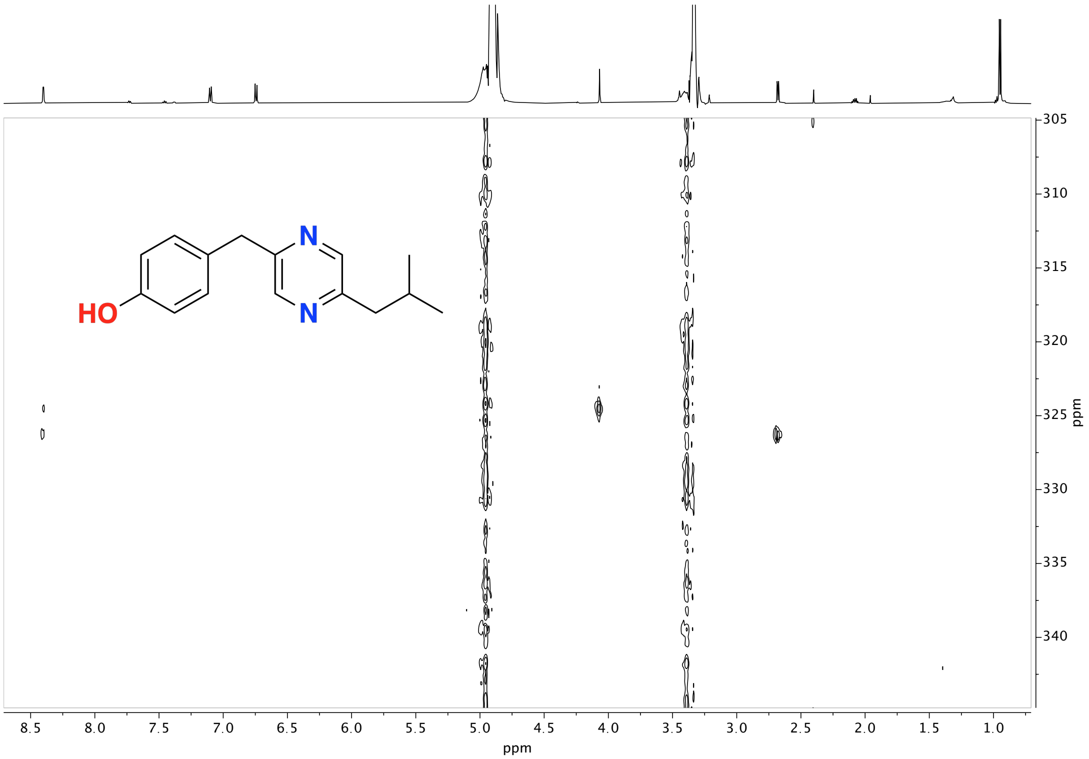

Figure S44. HRESI-MS spectrum of **6**

Elemental Composition Report **Page 1**

Single Mass Analysis
Tolerance = 5.0 mDa / DBE: min = -1.5, max = 100.0
Element prediction: Off
Number of isotope peaks used for i-FIT = 3

Monoisotopic Mass, Even Electron Ions
147 formula(e) evaluated with 2 results within limits (up to 50 closest results for each mass)
Elements Used:
C: 8-20 H: 0-150 N: 0-4 O: 0-40
HBL-02JAN2019-GA3-008-19-1 148 (2.520) AM2 (Ar,25000.0,0.00,0.00); ABS; Cm ((148+149))
TOF MS ES+

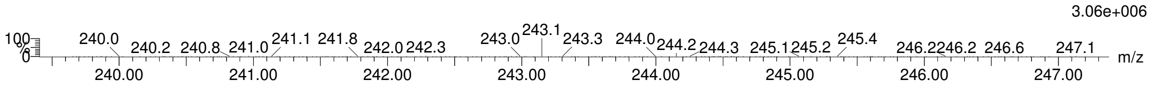

Mass	Calc. Mass	mDa	PPM	DBE	i-FIT	Norm	Conf(%)	Formula
Minimum:				-1.5				
Maximum:		5.0	5.0	100.0				
243.1496	243.1497	-0.1	-0.4	7.5	708.8	0.000	99.99	C15 H19 N2 O
	243.1457	3.9	16.0	3.5	718.2	9.463	0.01	C10 H19 N4 O3

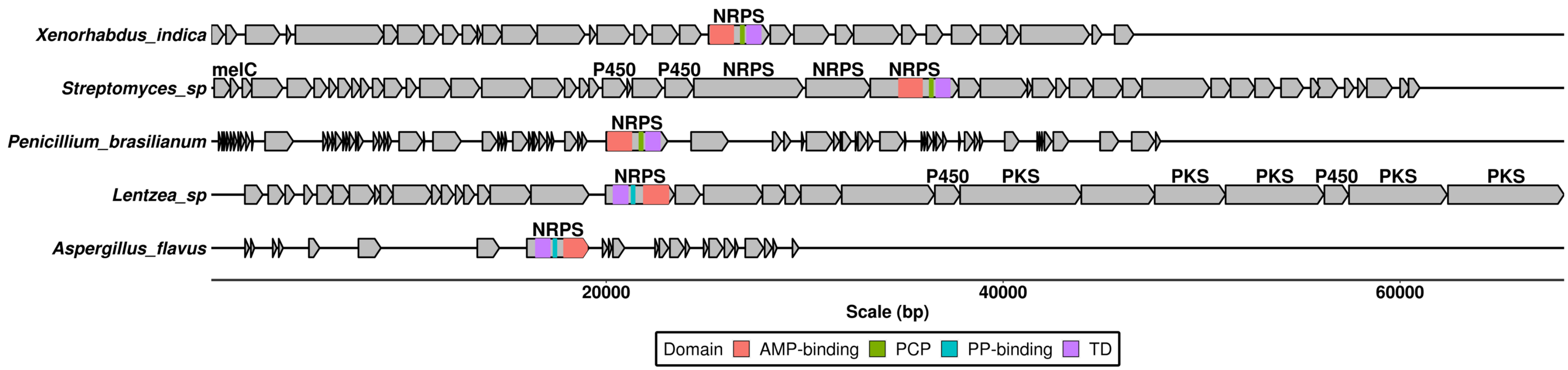

Figure S45. BGC structure of monomodular NRPSs associated with piperazines. BGCs are labelled per producing organism as described in *Lentzea* sp (this study), *Xenorhabdus indica*,[1] *Aspergillus flavus,*[2] *Streptomyces* sp.[3] and *Penicillium brasilianum*.[4] Peptidyl-carrier protein (PCP) and Phosphopantetheine (PP) domains and Terminal reductase domain (TD) are shown for monomodular NRPS-like gene.

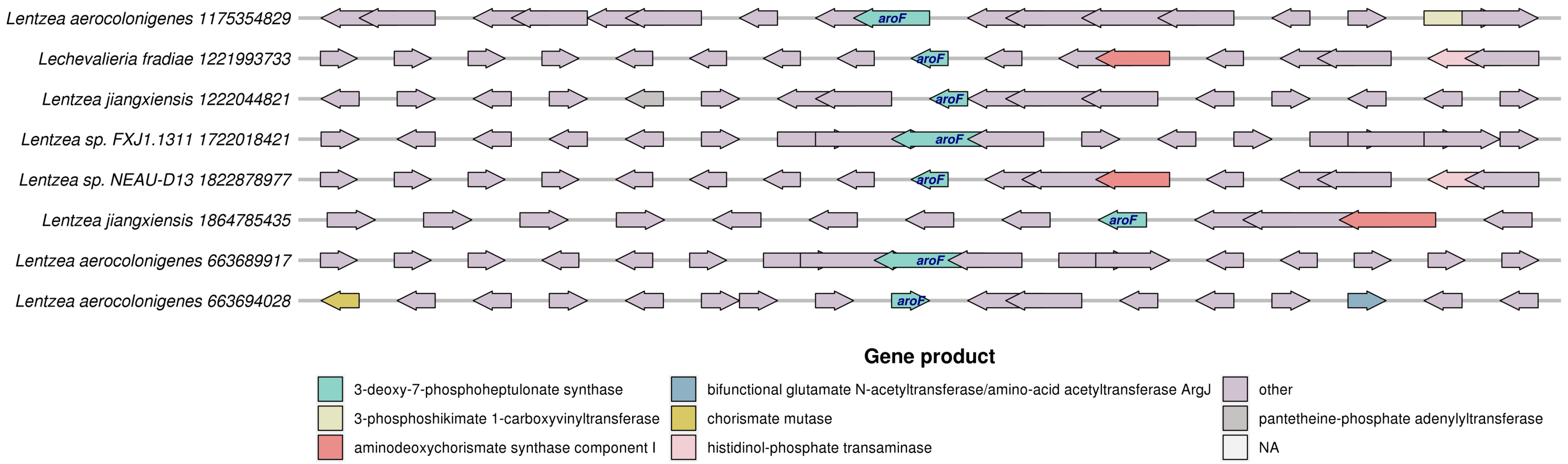

Figure S46. Location of aroF genes in *Lentzea* spp.
In *Lentzea* sp. GA3-008, the gene *KOEHFPPE_08076* is a homologue of Tyr-sensitive phospho-2-dehydro-3-deoxyheptonate aldolase, commonly annotated as *aroF* (EC:2.5.1.54), is adjacent to the single module NRPS *KOEHFPPE_08075* in GA3-008. A BLAST search of this gene sequence returns eight *Lentzea* spp. with aroF genes, all of which are located near other shikimate pathway genes on the chromosome.

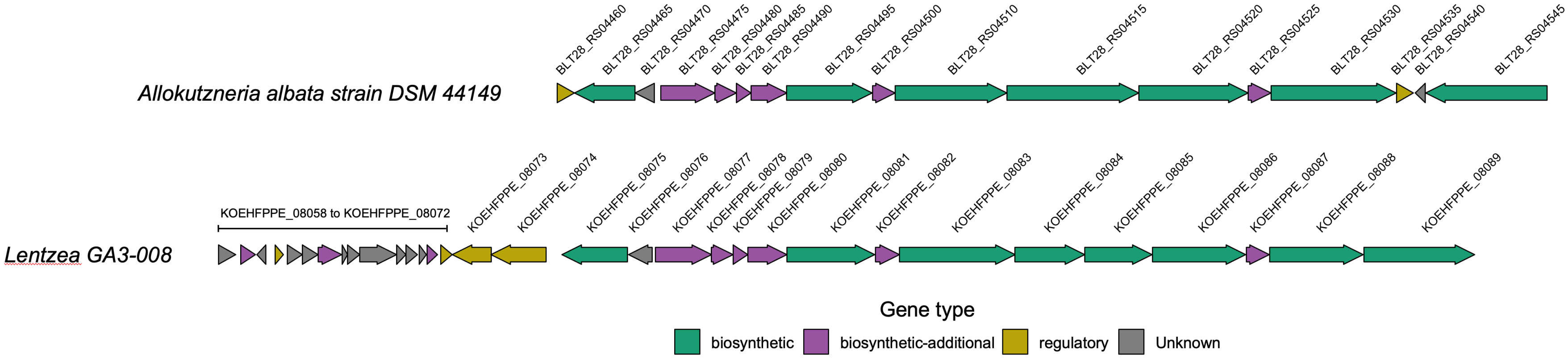

Figure S47. Comparison of monomodular NRPS genes in *Lentzea* sp. GA3-008 and *Allokutzneria albata* DSM 44149.

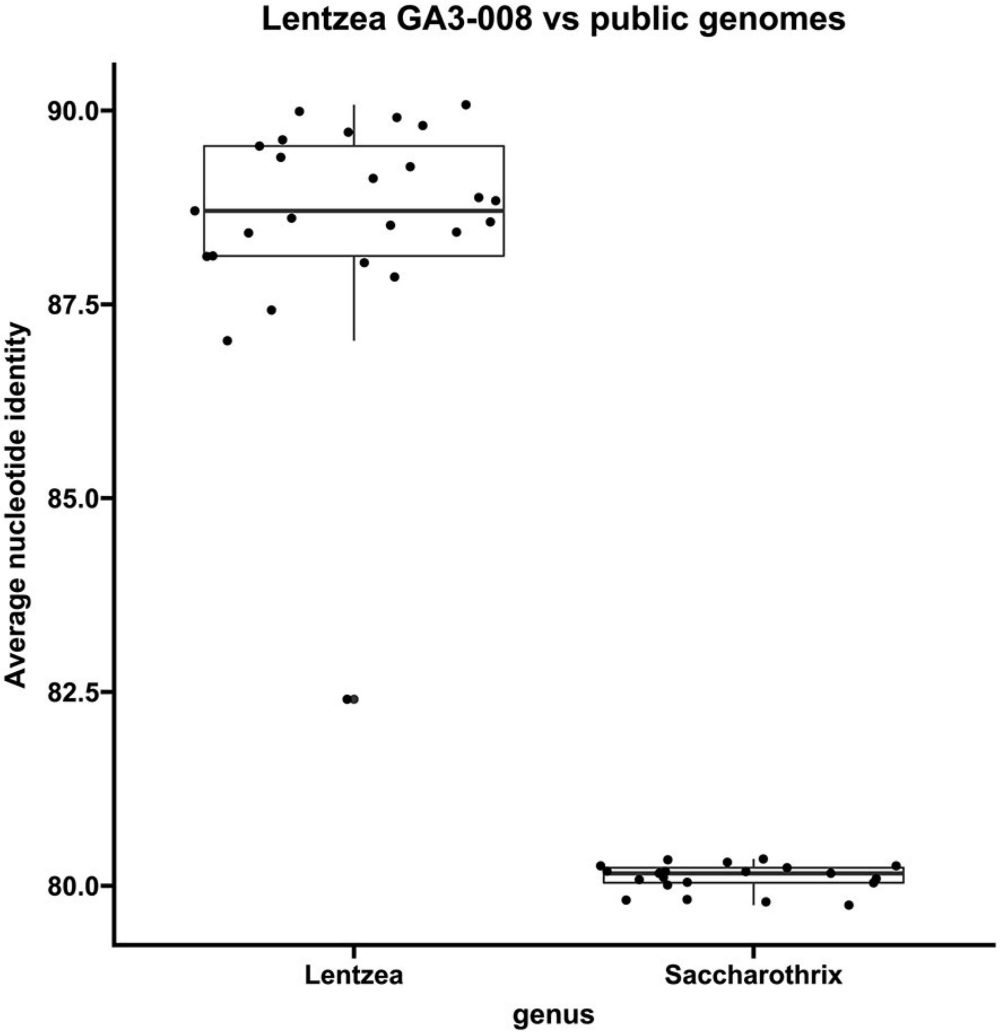

Figure S48. All Nucleotide Identities for *Lentzea* spp and *Saccharothrix* spp supporting the taxonomic assignment of GA3-008 to the genus *Lentzea.*

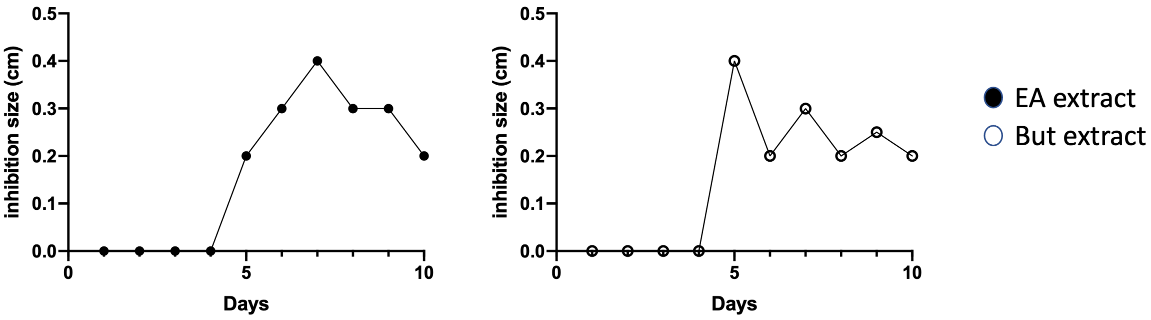

Figure S49. Antimicrobial activity as a function of time. Assays were conducted in a 96-well/pinhole format and zones of inhibition were measured after 16-18 hours growth. See Methods for experimental details.

Table S1. Biosynthetic gene clusters predicted in *Lentzea sp*. identified by antiSMASH.

Region	type	From	To	Most similar known cluster		similarity
1	PKS-like	33,068	72,248	marineosin A / B	Polyketide	9%
2	T1PKS	82,126	129,970	rifamorpholine A - E	Polyketide	7%
3	T1PKS	167,019	214,636	streptolydigin	NRP + Polyketide:Modular type I + Saccharide:Hybrid/tailoring	10%
4	arylpolyene	281,891	323,051	actagardine	RiPP:Lanthipeptide	6%
5	T3PKS	611,118	651,141	alkyl-O-dihydrogeranyl-methoxyhydroquinones	Terpene + Polyketide	57%
6	NRPS	827,666	875,738	coelichelin	NRP	72%
7	indole	998,404	1,019,375	staurosporine	Alkaloid	80%
8	lassopeptide	1,312,230	1,334,650	citrulassin D	RiPP	100%
9	terpene	1,347,055	1,365,171	SF2575	Polyketide:Type II + Saccharide:Hybrid/tailoring	6%
10	NRPS	1,578,089	1,669,269	coelibactin	NRP	36%
11	terpene	1,702,444	1,723,339	tiancilactone	Terpene	17%
12	terpene,siderophore	1,747,955	1,777,950	lysolipin I	Polyketide	4%
13	thiopeptide,LAP	2,399,750	2,424,467			
14	NRPS-like	3,022,590	3,065,211			
15	LAP,thiopeptide	4,344,514	4,373,342	cyclothiazomycin	RiPP:Thiopeptide	33%
16	NRPS,PKS-like,T1PKS	5,332,053	5,415,002	maklamicin	Polyketide	15%
17	terpene	5,416,624	5,436,010			
18	NRPS-like	5,461,971	5,505,044	indigoidine	NRP	80%
19	terpene	5,537,904	5,559,273	geosmin	Terpene	100%
20	lanthipeptide	6,097,862	6,119,025	Ery-3-9	RiPP:Lanthipeptide	75%
21	PKS-like,T2PKS	6,141,298	6,229,776	LL-D49194α1 (LLD)	Polyketide	39%
22	betalactone,terpene	6,232,300	6,272,242	microansamycin	Polyketide	7%
23	bacteriocin	6,462,100	6,472,738			
24	T3PKS	6,859,962	6,898,752			
25	lanthipeptide	6,959,864	6,981,036	jomthonic acid A - C	NRP	5%
26	melanin	6,981,058	6,991,462	asukamycin	Polyketide:Type II	3%
27	T1PKS,NRPS-like	7,005,356	7,062,199	bafilomycin B1	Polyketide:Modular type I	22%
28	NRPS-like	7,081,013	7,123,884	nenestatin	Polyketide	3%
29	thiopeptide,LAP	7,134,861	7,184,980	ECO-02301	Polyketide	7%
30	CDPS	7,318,387	7,339,067			
31	indole	7,405,929	7,427,074	fortimicin	Saccharide	6%
32	lanthipeptide	7,576,096	7,598,741			
33	terpene	7,692,284	7,712,308	geosmin	Terpene	100%
34	T1PKS	8,138,510	8,226,594	phoslactomycin B	Polyketide	35%
35	hglE-KS	8,428,540	8,477,841			
36	terpene	8,524,197	8,545,129	2-methylisoborneol	Terpene	50%
37	terpene	8,639,173	8,660,099	isorenieratene	Terpene	71%
38	NRPS	8,989,420	9,040,386	salinamide A	NRP	21%
39	T1PKS,T3PKS	9,064,908	9,128,678	A-94964	Other	59%
40	hglE-KS,T1PKS	9,154,261	9,199,325	paramagnetoquinone 1 / 2	Polyketide	10%

References

1. Tietze, A.; Shi, Y. N.; Kronenwerth, M.; Bode, H. B., Nonribosomal Peptides Produced by Minimal and Engineered Synthetases with Terminal Reductase Domains. *ChemBioChem* **2020,** *21*, 2750-2754.
2. Forseth, R. R.; Amaike, S.; Schwenk, D.; Affeldt, K. J.; Hoffmeister, D.; Schroeder, F. C.; Keller, N. P., Homologous NRPS-like Gene Clusters Mediate Redundant Small-Molecule Biosynthesis in Aspergillus flavus. *Angewandte Chemie International Edition* **2013,** *52*, 1590-1594.
3. Tanifuji, R.; Koketsu, K.; Takakura, M.; Asano, R.; Minami, A.; Oikawa, H.; Oguri, H., Chemo-enzymatic Total Syntheses of Jorunnamycin A, Saframycin A, and N-Fmoc Saframycin Y3. *Journal of the American Chemical Society* **2018,** *140*, 10705-10709.
4. Yuan, B.; Liu, D.; Guan, X.; Yan, Y.; Zhang, J.; Zhang, Y.; Yang, D.; Ma, M.; Lin, W., Piperazine ring formation by a single-module NRPS and cleavage by an α-KG-dependent nonheme iron dioxygenase in brasiliamide biosynthesis. *Applied Microbiology and Biotechnology* **2020,** *104*, 6149-6159.
